# Two local minima for structures of [4Fe–4S] clusters obtained with density functional theory methods

**DOI:** 10.1038/s41598-023-37755-0

**Published:** 2023-07-04

**Authors:** Sonia Jafari, Ulf Ryde, Mehdi Irani

**Affiliations:** 1 Department of Chemistry, University of Kurdistan, P.O.Box 66175-416, Sanandaj, Iran; 2 Department of Theoretical Chemistry, Lund University, P.O.Box 124, 221 00 Lund, Sweden

**Keywords:** Biochemistry, Computational biology and bioinformatics, Chemistry

## Abstract

[4Fe–4S] clusters are essential cofactors in many proteins involved in biological redox-active processes. Density functional theory (DFT) methods are widely used to study these clusters. Previous investigations have indicated that there exist two local minima for these clusters in proteins. We perform a detailed study of these minima in five proteins and two oxidation states, using combined quantum mechanical and molecular mechanical (QM/MM) methods. We show that one local minimum (L state) has longer Fe–Fe distances than the other (S state), and that the L state is more stable for all cases studied. We also show that some DFT methods may only obtain the L state, while others may obtain both states. Our work provides new insights into the structural diversity and stability of [4Fe–4S] clusters in proteins, and highlights the importance of reliable DFT methods and geometry optimization. We recommend r^2^SCAN for optimizing [4Fe-4S] clusters in proteins, which gives the most accurate structures for the five proteins studied.

## Introduction

Direct electron transfer occurs in biological systems via iron–sulfur clusters, cytochromes, and blue copper proteins^1^. Iron–sulfur clusters were identified about 50 years ago in biological systems^2^. Since then, it has become evident that they play many important roles in biology. They are common in nature and are essential for electron transport^3^ and catalysis^4^. These roles often overlap with oxidoreductase proteins, which catalyze electron and proton transfers, as well as substrate binding and catalytic changes^5–8^.

**Figure 1:**
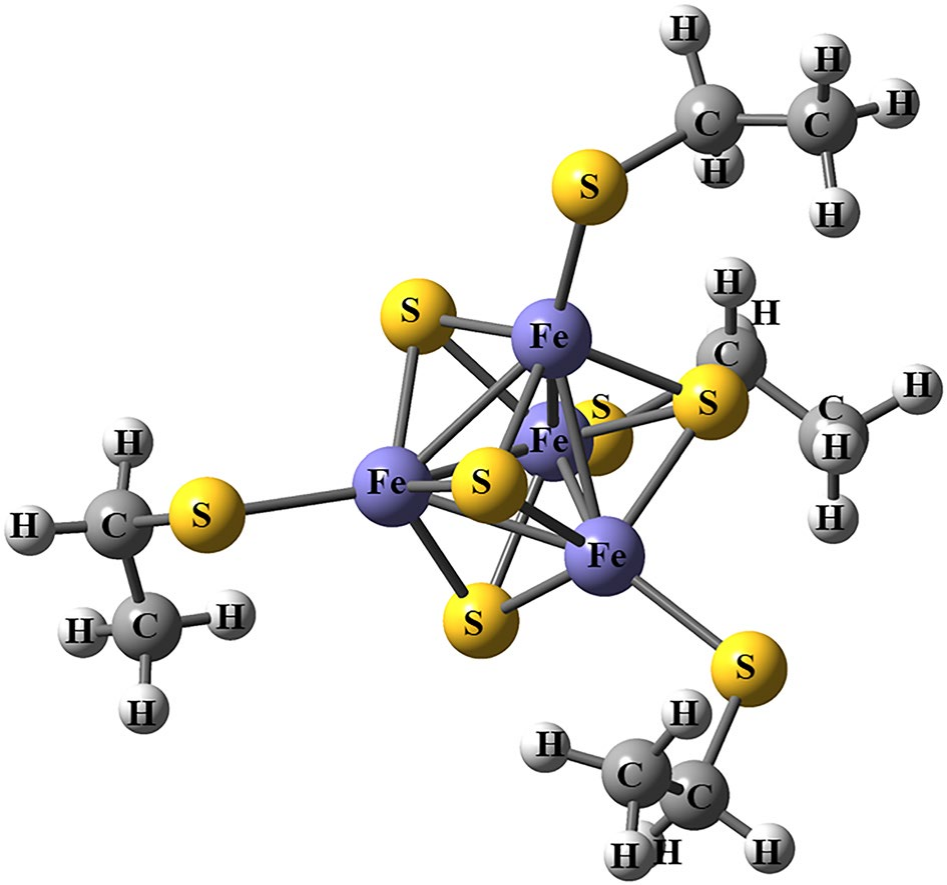
QM system in the QM/MM calculations.

There are several types of FeS sites. Rubredoxins contain the simplest FeS site, which consists of a single Fe ion coordinated to four cysteine (Cys) residues^9,10^. The [2Fe–2S] ferredoxins have two Fe ions, two bridging sulfide ions, and two Cys residues coordinated to each Fe ion^11,12^. The Rieske site has another type of [2Fe–2S] cluster in which two histidine (His) residues coordinate one of the Fe ions in place of Cys^13^. Some proteins contain more complicated [4Fe–4S] clusters^14–16^, which comprise four Fe ions that are connected by four sulfide ions. In addition, each Fe ion is coordinated to a Cys residue^17^ (cf. Fig. 1). There are also ferredoxins with [3Fe–4S] clusters^14,18^, in which one Fe ion and one Cys residue are missing compared to [4Fe–4S] ferredoxins. More complicated iron–sulfur clusters are found in some proteins (such as the Fe_8_S_7_Cys_6_ P-cluster and the MoFe_7_S_9_C FeMo cluster in nitrogenase), are sometimes associated with catalytic functions^19,20^.

The [4Fe–4S] clusters in ferredoxins use the ${\mathrm{Fe}}_{3}^{\mathrm{II}}{\mathrm{Fe}}_{1}^{\mathrm{III}}/{\mathrm{Fe}}_{2}^{\mathrm{II}}{\mathrm{Fe}}_{2}^{\mathrm{III}}$ redox couple, which results in redox potentials between − 0.7 and − 0.3 V^21^. High-potential iron–sulfur proteins (HiPIP) also contain [4Fe–4S] clusters, but in contrast to the [4Fe–4S] ferredoxins, they utilize ${\mathrm{Fe}}_{2}^{\mathrm{II}}{\mathrm{Fe}}_{2}^{\mathrm{III}}/{\mathrm{Fe}}_{1}^{\mathrm{II}}{\mathrm{Fe}}_{3}^{\mathrm{III}}$ redox pair, giving them a substantially more positive potential (+ 0.05 to + 0.5 V). Several spectroscopic, structural, and theoretical methods have been used to study the physical characteristics and electronic structures of FeS clusters^22^. Synthetic analogs of the FeS clusters have also been intensively studied^23^.

In recent years, computational chemistry calculations have been widely used to evaluate and predict structural and electrical properties of transition metal complexes^22,24–35^. Most of these studies have been performed with density functional theory (DFT).

Systems with several spin-coupled metal ions have complicated electronic structures. In the [4Fe–4S] clusters, the individual iron ions are typically in the high-spin state, but these spins are antiferromagnetically coupled to a lower total spin. It is crucial to characterize the antiferromagnetic coupling at the same level of theory as the strong metal–ligand bonding and the weaker metal–metal interactions to discuss trends in [4Fe–4S] clusters. The broken-symmetry (BS) approach^36,37^, which considers weakly interacting electrons physically, makes this possible.

Accurate geometrical structures are important to predict the electronic properties of iron–sulfur clusters. Case et al.^35^ used DFT to calculate redox potentials for a few iron–sulfur clusters. Later, they extended their work using a similar method but with optimized structures of the clusters^22^ rather than assumed geometries. The results indicated that optimized structures tend to give longer bond lengths than the experimentally observed ones, e.g., by 0.03 Å for the [Fe_4_S_4_(SCH_3_)_4_]^2−^ model cluster. This study also better reproduced the experimentally determined redox potentials, which they partly attributed to the geometry optimization.

Recently, we systematically investigated the redox potentials of iron–sulfur clusters with various quantum mechanics/molecular mechanics (QM/MM) and QM-cluster methods^24^. We then observed conspicuous differences in the Fe–Fe distances for some of the QM/MM structures for [4Fe–4S] clusters in proteins. Since the geometry plays a significant role in the accuracy of redox calculations, we decided to investigate this observation in depth. In this work, we investigate the occurrence and nature of two local minima for [4Fe–4S] clusters in proteins using QM/MM methods with various DFT functions and basis sets. We focus on five proteins that contain [4Fe–4S] clusters in different oxidation and spin states: three ferredoxins and two HiPIPs. We show that one local minimum (L state) has longer Fe–Fe distances than the other (S state). We also show that some DFT methods may only obtain the L state, while others may obtain both states. We compare the local minima and discuss their implications for computational models. Our work provides new insights into the structural diversity and stability of [4Fe–4S] clusters in proteins, and highlights the importance of using reliable DFT methods for accurate modeling of these systems. Additionally, we investigate how the local minima affect the BS states. The paper is organized as follows: “Methods” describes the computational methods and models used; “Result and discussion” presents the results and discussion; “Conclusions” summarizes the main conclusions and perspectives.

## Methods

### Studied systems

We have studied five [4Fe–4S] clusters in protein crystal structures. The proteins and the employed crystal structures are described in Table 1. Three [4Fe–4S] ferredoxins were studied from *Bacillus thermoproteolyticus* (1IQZ; 4Fd1)^15^, *Desulfovibrio africanus* (1FXR; 4Fd2)^16^, and *Azotobacter vinelandii* (5FD1; 4Fd3)^14^. In addition, two HiPIP sites were studied from *Allochromatium vinosum* (1CKU; Hip1)^38^ and *Halorhodospira halophila* (2HIP; Hip2). All QM/MM structures were taken from our recent study^24^, in which a description of the setup of the proteins, the protonation states, and the equilibration of the structure can be found.

**Table 1:** Studied systems, describing the FeS site, the source, the abbreviation used in the article (Abb), the crystal structure used for the calculations (protein databank code; PDB) and the resolution (*Res*) in Å, the number of Fe(II) ions (formally) in the reduced state (*n*^II^_red_), as well as the spin state for the reduced and oxidized states (*S*_red_ and *S*_ox_).

Site	Organism	Abb	PDB	*Res*	*n*^II^_red_	*S*_red_	*S*_ox_
[4Fe–4S] ferredoxin	*Bacillus thermoproteolyticus*	4Fd1	1IQZ^15^	0.92	3	1/2	0
	*Desulfovibrio africanus*	4Fd2	1FXR^16^	2.3	3	1/2	0
	*Azotobacter vinelandii*	4Fd3	5FD1^14^	1.9	3	1/2	0
HiPIP	*Allochromatium vinosum*	Hip1	1CKU^38^	1.2	2	0	1/2
	*Halorhodospira halophila*	Hip2	2HIP^39^	2.5	2	0	1/2

### QM calculations

QM calculations were performed using the Turbomole software^40^. We employed nine different DFT methods: four GGA functionals, with no admixture of HF exchange: PBE^41^, BP86^42,43^, BLYP^42,44^, and B97D^45^, two meta GGA functional with no HF exchange: TPSS^46^ and r^2^SCAN^47^, as well as three hybrid functionals: TPSSh (10% HF exchange)^48^, B3LYP (20% HF exchange^42,44,49^, and B3LYP* (15% HF exchange)^42,44,49,50^. All the functionals were combined with two different basis sets (def2-SV(P)^51^ or def2-TZVPD^52,53^). The calculations were sped up by the resolution-of-identity approximation^54–56^ which is a variational fitting of the electron density in an auxiliary basis set (we employed the built-in def2-SV(P)^51^ and universal^57^ auxiliary basis sets in Turbomole). Empirical dispersion corrections were included with the DFT-D3 approach^58^ and Becke–Johnson damping^59^, as implemented in Turbomole.

In some calculations, the QM system was immersed into a continuum solvent, employing the conductor-like screening model (COSMO)^60,61^. The default optimized COSMO atomic radii and a water solvent radius of 1.3 Å were employed to construct the solvent-accessible surface cavity^62^. For the Fe ions, a radius of 2.0 Å was used^63^. Structures for the QM + COSMO calculations were taken directly from the QM/MM calculations without further optimization. The dielectric constant of proteins has been much discussed, but values of 4–20 are typically used^64,65^. We tested three values for the dielectric constant (4, 20, and 80).

The QM system consisted of the Fe and S ions, as well as the directly coordinated Cys groups, modeled by CH_3_CH_2_S^–^, i.e., Fe_4_S_4_(SCH_2_CH_3_)_4_ for the whole cluster (cf. Fig. 1).

The electronic structures of the iron–sulfur clusters are complicated. Each Fe ion is in the high-spin state (five and four unpaired electrons for Fe(III) and Fe(II), respectively). However, these spins are coupled antiferromagnetically to a lower spin state in the polynuclear clusters, *S* = 0 or ½^66,67^, as is specified in Table 1. The BS approach in DFT calculations describes such antiferromagnetically coupled sites^36,37^. There are six possible BS states for the [4Fe–4S] clusters (two Fe ions with dominant beta spin can be selected among the four Fe ions in six different ways). We examined all possibilities and selected the one with the most favorable energy for the QM system of each protein and oxidation state with TPSS/def2-SV(P). This BS state was also used for the other calculations. In general, we discuss results only for the energetically lowest BS state.

The BS states were generated either by the fragment approach of Szilagyi and Winslow^33^ or by obtaining one BS state by first optimizing the highest possible spin state (all unpaired electrons aligned), flipping the spins to the desired state, and then obtaining the other BS states by simply swapping coordinates of the Fe ions^68^. Spin densities, geometries and relative energies of the various BS states for the two oxidation states of 4Fd3 at the PBE/def2-SV(P) level of theory are shown in Supplementary Tables S12–S14 in the Supplementary Information.

### QM/MM calculations

The QM/MM calculations were performed with the ComQum software^69,70^. In this approach, the protein and solvent are split into two subsystems: System 1 (the QM region) was relaxed by the QM method, whereas system 2 involved the remaining part of the protein and the solvent, and was kept fixed at the original coordinates (equilibrated crystal structure). In the QM calculations, system 1 was represented by a wavefunction (the functionals and basis sets are described in the previous section), whereas all the other atoms were represented by an array of partial point charges, one for each atom, taken from the MM setup. Thereby, the polarization of the QM system by the surroundings is included in a self-consistent manner. When there is a bond between systems 1 and 2, the hydrogen link-atom approach was employed: The QM system was capped with hydrogen atoms (hydrogen link atoms, HL), the positions of which are linearly related to the corresponding carbon atoms (carbon link atoms, CL) in the full system^69,71^. All atoms were included in the point-charge model, except the CL atoms^72^. Further details of the QM/MM calculations are given in the Supplementary Information.

## Results and discussion

This study examines the structure of [4Fe–4S] clusters in proteins. The test set includes three [4Fe–4S] ferredoxins and two high-potential iron–sulfur proteins (cf. Table 1). In our previous study, we observed extensive differences in the Fe–Fe distances of the clusters in different proteins when optimizing the structures at the TPSS/def2-SV(P) level of theory. After some test calculations, we found out that the [4Fe–4S] clusters in all five proteins and all studied oxidation states could attain two local minima. Consequently, we have systematically studied the energies and electronic properties of these two minima. The two local minima were obtained with the QM/MM calculations, either by first restraining some Fe–Fe distances in initial optimizations and then removing all restraints and reoptimizing the structure or simply by starting from the other oxidation state (when they represent different minima). In the following, we analyze the geometry of the two local minima in both oxidation states, their spin states, and how their stability varies with the DFT functionals and basis sets. Furthermore, we examine how the environment (modeled either by point charges in QM/MM calculations or implicitly by a continuum solvent) affects the stability of the minima. We also test whether the minima can be obtained with other functionals and basis sets besides TPSS/def2-SV(P).

### Geometries

Table 2 shows a statistical analysis of the calculated Fe–Fe and Fe–S distances for the five studied FeS clusters at the TPSS/def2-SV(P) level of theory (the raw data are given in Supplementary Tables S1–S5 in the Supplementary Information). It can be seen that the DFT calculations give two local minima for all structures and oxidation states. They differ primarily in the Fe–Fe distances. For example, for the Ox state of 4Fd1, one local minimum has Fe–Fe distances of 2.76–2.80 Å (2.77 Å on average), whereas for the other, the distances are 2.56–2.66 Å (2.64 Å on average), i.e. a difference of 0.13 Å on average. In the following, these local minima will be referred to as L and S (long and short). From Table 2, it can be seen that the average Fe–Fe distances are the same for all ferredoxin sites: 2.76–2.78 vs. 2.63–2.65 Å for L and S, respectively (i.e. a difference of 0.12–0.13 Å between L and S). The reduced sites have only 0.01 Å shorter average Fe–Fe distances. The reduced HiPiPs, also have similar Fe–Fe distances, but for the oxidized HiPIPs the Fe–Fe distances are 2.80–2.81 vs. 2.58 Å, with an appreciably larger difference between L and S (0.22–0.23 Å) and also a larger difference between the two oxidation states (0.02–0.04 Å; in fact, the average Fe–Fe distance increase upon reduction for the S states). This is caused by the differing ${\mathrm{Fe}}_{1}^{\mathrm{II}}{\mathrm{Fe}}_{3}^{\mathrm{III}}$ oxidation state.

**Table 2:** Average of Fe–Fe (Av_Fe–Fe_) and Fe–S (Av_Fe–S_) distances, as well as the mean absolute deviations (MADs) from the corresponding crystal structures of the Fe–Fe (MAD_Fe–Fe_) and Fe–S (MAD_Fe–S_) distances for the five studied FeS clusters. The values are in Å and are from the TPSS/def2-SV(P) optimized structures. The raw data can be found in Supplementary Tables S1–S5 in the Supplementary Information.

	Av_Fe–Fe_					Av_Fe–S_					MAD_Fe–Fe_				MAD_Fe–S_			
	Crystal	Ox		Red		Crystal	Ox		Red		Ox		Red		Ox		Red	
		L	S	L	S		L	S	L	S	L	S	L	S	L	S	L	S
4Fd1	2.73	2.77	2.64	2.76	2.63	2.29	2.31	2.30	2.34	2.32	0.05	0.09	0.04	0.10	0.03	0.03	0.05	0.04
4Fd2	2.73	2.77	2.65	2.76	2.64	2.27	2.32	2.30	2.34	2.33	0.04	0.09	0.03	0.10	0.06	0.05	0.08	0.07
4Fd3	2.71	2.78	2.65	2.77	2.65	2.29	2.31	2.30	2.33	2.32	0.06	0.06	0.06	0.06	0.05	0.04	0.05	0.04
Hip1	2.72	2.80	2.58	2.78	2.62	2.28	2.29	2.25	2.31	2.29	0.08	0.14	0.06	0.10	0.04	0.04	0.03	0.02
Hip2	2.66	2.81	2.58	2.77	2.62	2.21	2.29	2.25	2.32	2.30	0.15	0.07	0.12	0.04	0.11	0.07	0.12	0.10

Table 2 shows also the average Fe–S distances. They show appreciably smaller differences between the L and S local minima: 0.01–0.02 Å for the ferredoxins and reduced HiPIPs, but 0.04 Å for the oxidized HiPIPs (L always gives longer average Fe–S distances). The average Fe–S distances increase upon reduction by 0.02–0.05 Å for all sites and minima (most for S of the HiPIPs).

Naturally, it is interesting to know which of the two local minima agrees best with the crystal structures of the corresponding proteins. In Table 2, we show the average Fe–Fe and Fe–S distances in the crystal structures, as well as the mean absolute difference of the Fe–Fe (MAD_Fe–Fe_) and Fe–S distances (MAD_Fe–S_) from the crystal values for the four optimized structures of each protein. It can be seen that the crystallographic Fe–Fe distances are always in between those obtained for the two minima, 2.66–2.73 Å. For three of the proteins, the reduced L minimum gives the smallest MAD_Fe–Fe_ (0.03–0.06 Å) among the four optimized structures, whereas for Hip2, instead Ox–S gives the best results and for 4Fd3, all four structures give the same MAD. For MAD_Fe–S_, instead, Ox–S gives the best results for four of the structures (0.03–0.07 Å) and Red–S for Hip1. However, the differences are small and in two cases, other structures (Ox–L or Red–S) give the same MAD. Thus, it seems hard to point out which of the two minima correspond to the crystal structures, probably owing to the mediocre accuracy of the crystal structures and the risk that they are partly photoreduced during crystallography and therefore may represent a mixture of oxidation states.

### Stability of the local minima with different methods

Next, we calculated the relative stability of the two local minima in the two oxidation states and the five proteins. From the results in Table 3, it can be seen that the L state is always more stable than the S state, by 26–30 kJ/mol for the ferredoxins and by 19–24 kJ/mol for the HiPIPs (3 kJ/mol less for the Ox state than for the Red state).

**Table 3:** Relative energies of the local DFT minima in kJ/mol, defined as the difference between the L and S structures for the same redox state (i.e. *E*(**S**)—*E*(**L**)). All structures were optimized by the QM/MM method at the TPSS/def2-SV(P) level of theory. The other results are single-point calculations on these using other DFT methods (B3LYP), basis sets (def2-TZVPD), or with a COSMO continuum solvation model at dielectric constants of 4, 20, and 80.

	QM/MM						COSMO-TPSS/def2-SV(P)					
	TPSS/def-SV(P)		B3LYP/def2-SV(P)		TPSS/def2-TZVPD		ε = 4		ε = 20		ε = 80	
	Ox	Red	Ox	Red	Ox	Red	Ox	Red	Ox	Red	Ox	Red
4Fd1	27.0	26.0	88.2	91.2	17.3	29.4	27.0	25.0	26.1	23.1	25.9	22.7
4Fd2	28.9	27.8	86.0	88.8	12.3	27.3	28.2	29.1	27.7	28.4	27.5	28.4
4Fd3	29.7	30.0	87.6	91.0	12.0	14.8	27.6	27.1	27.2	25.8	27.1	25.5
Hip1	18.8	22.3	151.1	93.6	2.2	42.7	18.3	22.0	18.2	21.4	18.2	21.2
Hip2	20.1	22.9	158.8	93.6	22.7	26.8	22.5	23.8	22.3	23.0	22.3	22.9

We also performed single-point energy calculations on the TPSS/def2-SV(P)-optimized structures of the two local minima for all systems, changing the functional to B3LYP (with the def2-SV(P) basis set), changing the basis set to def2-TZVPD (with the TPSS functional), and changing the explicit QM/MM environment of the [4Fe–4S] cluster to an implicit one (COSMO continuum solvation model with dielectric constants of 4, 20, or 80; TPSS/def2-SV(P) calculations). The results presented in Table 3 show that the description of the surroundings has a very small effect, changing the relative stabilities by less than 5 kJ/mol. Likewise, the three dielectric constants give the same relative energies within 2 kJ/mol. On the other hand, the basis set gives a larger effect, up to 20 kJ/mol. Moreover, B3LYP increases the stability of the L minimum (to 86–94 kJ/mol, but 151–159 for the Ox state of the HiPIPs, compared to 19–30 kJ/mol with the TPSS).

### Spin states of the local minima

Mulliken spin populations of the iron ions in the various oxidation states and local minima at the TPSS/def2-SV(P) level of theory are collected in Table 4. It can be seen that for the sites with the ${\mathrm{Fe}}_{2}^{\mathrm{II}}{\mathrm{Fe}}_{2}^{\mathrm{III}}$ charge state (Ox 4Fd and Red HiPIP), the Fe spin populations are the same on all four Fe ions (within 0.03–0.09 *e*), 3.4–3.5 *e* for the L minimum and 3.3–3.4 *e* for the S minimum (in absolute terms). Thus, the Fe spin populations of the S local minimum are always slightly lower than those of the L minimum, by 0.13 *e* on average. For the other two charge states, two of the Fe ions (those with a surplus of β spin, i.e. a negative spin population in Table 4) have a larger spin population, and the other two have a lower spin population, with a difference of 0.2–0.5 *e*. For the Red 4Fd sites, the spin populations are 2.9–3.6 *e*, still with a difference of 0.1–0.2 *e* between the two minima. However, for the oxidized state of the two HiPIPs, the difference is much larger, 0.4–0.8 *e* with an average of 0.53 *e*, because the S minimum has smaller spin populations, 3.1 *e* for those with negative populations and 2.6–2.9 *e*, for those with positive populations. It can also be seen that the L and S local minima do not depend on the BS states (i.e., which Fe ions have negative spin populations): The various proteins have different preferred BS states, but both local minima are found for all proteins, obtained for the same BS state for each protein. Enlarging the basis set from def2-SV(P) to def2-TZVPD has a small effect on the spin populations. Further details of spin populations with the B3LYP functional and the def2-TZVPD basis set are given in the Supplementary Information.

**Table 4:** Mulliken spin populations of the iron ions in the two local minima at the TPSS/def2-SV(P) level of theory for the five FeS protein and the two oxidation states.

		Ox-L	Ox-S	Red-L	Red-S
4Fd1	Fe1	− 3.5	− 3.4	− 3.5	− 3.4
	Fe2	3.5	3.4	3.3	3.3
	Fe3	− 3.5	− 3.3	− 3.6	− 3.5
	Fe4	3.5	3.4	3.2	3.0
4Fd2	Fe1	3.5	3.4	3.4	3.3
	Fe2	− 3.4	− 3.3	− 3.6	− 3.5
	Fe3	3.5	3.4	3.1	2.9
	Fe4	− 3.5	− 3.4	− 3.6	− 3.5
4Fd3	Fe1	− 3.5	− 3.4	− 3.5	− 3.4
	Fe2	3.4	3.3	3.3	3.2
	Fe3	− 3.5	− 3.3	− 3.6	− 3.5
	Fe4	3.5	3.4	3.3	3.1
Hip1	Fe1	− 3.6	− 3.1	− 3.5	− 3.3
	Fe2	3.3	2.6	3.5	3.3
	Fe3	− 3.5	− 3.1	− 3.4	− 3.3
	Fe4	3.3	2.8	3.4	3.3
Hip2	Fe1	3.3	2.9	3.5	3.3
	Fe2	3.3	2.6	3.5	3.3
	Fe3	− 3.6	− 3.1	− 3.5	− 3.3
	Fe4	− 3.6	− 3.1	− 3.5	− 3.3

### Dependence of the S and L local minima on the DFT functional and basis set

The results presented above are based on structures optimized at the TPSS/def2-SV(P) level of theory. In this section, we consider structures optimized with other DFT functionals or basis sets, testing also the larger def2-TZVPD basis set^52,53^. We used the PBE^41^, BP86^42,43^, BLYP^42,44^, and B97-D^45^, functionals with no HF exchange, as well as the meta GGA TPSS^46^ and r^2^SCAN^47^ functionals, and the hybrid TPSSh (10% HF exchange)^48^, B3LYP (20% HF exchange)^42,44,49^, B3LYP* (15% HF exchange)^42,44,49,50^ and PBE0^73,74^ (25% HF exchange) functionals. Interestingly, we were able to reproduce the two local minima for some combinations of the functionals and basis sets, but for many combinations, the L local minimum was only obtained and S local minimum disappeared (structures optimized starting from the S minimum converged to the L minimum, indicating that S is converted from a local minimum to a shoulder on the potential energy surface). Table 5 summarizes the difference between Av_Fe–Fe_ values in the L and S states (Δ*D*_Fe–Fe_) as well as the energy difference between the L and S states (Δ*E* = *E*_**S**_ – *E*_**L**_) for all combinations of the functionals and the basis sets. As can be seen, the TPSS, BLYP, PBE, and BP86 pure functionals with the def2-SV(P) basis set give both local minima for all proteins in both the Red and Ox states. On the other hand, B97-D/def2-SV(P) gives only the L local minima, except for the Red states of Hip1 and Hip2. Moreover, the r^2^SCAN, TPSSh, B3LYP, and B3LYP* functionals give only the L minima for all proteins and redox states. As the basis set is expanded to def2-TZVPD, the S local minimum disappears for most functionals. Only some of the pure functionals (especially BP86 and PBE) give both local minima for some states, whereas the hybrid functionals and r^2^SCAN never show the S local minima with the larger basis set.

**Table 5:**
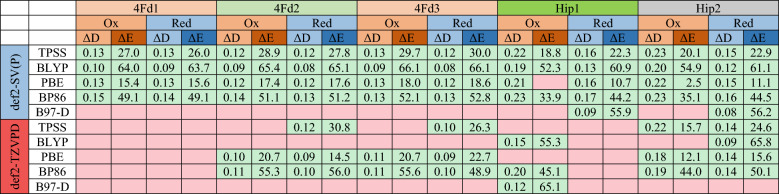
The difference between Av_Fe–Fe_ values in the L and S states (Δ*D*_Fe–Fe_) as well as the energy difference between the L and S states (Δ*E* = *E*_**S**_ – *E*_**L**_), when using all combinations of the functionals and the basis sets. Green cells indicate that the state has the two local minima with the identified Δ*D*_Fe–Fe_ and Δ*E* values. The red cells indicate that only the L local minimum was found for that state. For the r^2^SCAN, TPSSh, B3LYP, and B3LYP* functionals, no S local minimum was found for any system, so they are not included in the table.

It can be seen that the four functionals with results for all proteins with the small basis set give quite similar Δ*D*_Fe–Fe_ (slightly lower for BLYP than for the other three functionals) and also similar trends among the five proteins. For the proteins and functionals that give both minima for the large basis set, the increase in the basis set typically leads to a slightly smaller Δ*D*_Fe–Fe_ (by up to 0.04 Å). However, for ∆*E* the variation is larger. BLYP gives the largest values (52–66 kJ/mol), whereas PBE gives the smallest values (up to 19 kJ/mol), actually suggesting that for the two Ox HiPiP sites, the two minima are essentially degenerate. Still, the trends among the five proteins are the same. Increasing the basis sets typically increases ∆*E*, but the effect is small (− 4 to 11 kJ/mol).

Since the L local minimum is more stable than the S local minimum in both oxidation states for all enzymes and in all combinations of the functionals and basis sets, as well as being the only possible minimum for some functionals, we compared the calculated structures of L minima with the crystal structures. The raw data are shown in Tables S7–S11 in the Supplementary Information and the results are summarized in Tables 6 and 7, showing MAD_Fe–Fe_ and MAD_Fe–S_ for all proteins and oxidation states and all functionals and basis sets. To compare the various approaches, we averaged the lowest MAD value for the two oxidation states for each protein (the oxidation state of all crystal structures is not always reported and it may change during data collection owing to photoreduction). Based on these values for the Fe–Fe distances, it can be seen that r^2^SCAN/def2-SV(P) performs best with an average deviation of only 0.04 Å. The same functional with the larger basis set and TPSS, BLYP and PBE with the small basis set all have average deviations of 0.06 Å. Increasing the basis set deteriorates the results for all functionals (by 0.01 Å on average), indicating some cancellation of errors. B3LYP and B3LYP* give appreciably worse results than the other functionals (0.12–0.13 Å), whereas TPSSh gives results similar to those of the worst pure functionals. It is notable that Hip2 (the protein with the lowest resolution) gives twice as high MAD_Fe–Fe_ as the other proteins, 0.13 Å, compared to 0.06–0.08 Å (averaged over all functionals and basis sets).

**Table 6:**
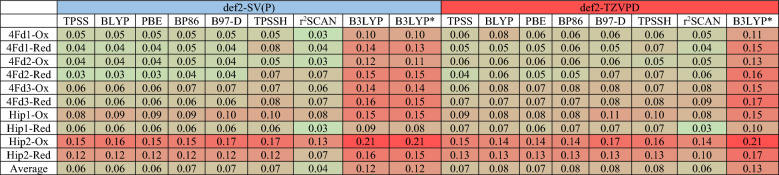
MAD_Fe–Fe_ values for the L local minima of the Ox and Red states of the proteins using the TPSS, BLYP, PBE, BP86, B97-D, TPSSH, r2SCAN, B3LYP and B3LYP* functionals, and def2-SV(P) and def2-TZVPD basis sets. The last row shows the average MAD_Fe–Fe_ values for a given level of theory. The values are all in Å.

**Table 7:**
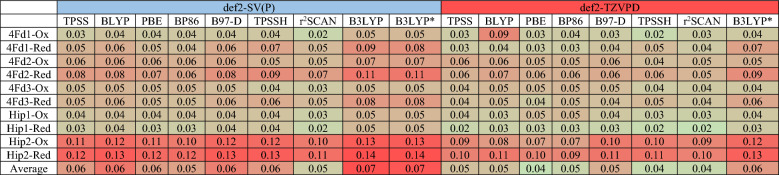
MAD_Fe–S_ values for the L local minima of the Ox and Red states of the proteins using the TPSS, BLYP, PBE, BP86, B97-D, TPSSH, r^2^SCAN, B3LYP and B3LYP* functionals, and def2-SV(P) and def2-TZVPD basis sets. The last row shows the average MAD_Fe–S_ values for a given level of theory. The values are all in Å.

The results for the Fe–S bond lengths are quite different. In this case, optimizations with the larger basis set give the best results (improving the average MADs by 0.01 Å on average). The lowest average MAD (0.04 Å) is obtained for PBE, TPSSh and r^2^SCAN. B3LYP and B3LYP* still give the worst results (0.06–0.07 Å), but the differences are small. Again, Hip2 gives appreciably worse MADs than the other four structures (0.10 compared to 0.03–0.06 Å, averaged over all DFT methods). Averaging the results for the two sets of distances indicates that r^2^SCAN with both basis sets gives the best result (0.05 Å), but the differences are small (all the other methods, except B3LYP and B3LYP* give an average MAD of 0.06 Å).

## Conclusions

Geometries are important for predicting accurate electronic properties of molecular systems and iron-sulfur clusters, and today DFT methods are widely used to evaluate and predict the geometrical and electronic properties of transition metal complexes. For example, DFT-optimized structures for a [Fe_4_S_4_(SCH_3_)_4_]^2–^ model cluster reproduce experimentally determined redox potentials more accurately than experimental structures^22^. In this study, we examined the recent observation that QM/MM calculations for [4Fe–4S] clusters in proteins may give two local minima at the differing in the Fe–Fe distances^24^. Accordingly, we have studied five [4Fe–4S] clusters in protein crystal structures shown in Table 1. We examined the geometry of local minima in both oxidation states, their spin states, and how their stability is affected by the choice of DFT functionals and basis sets.

The results indicate that the crystallographic Fe–Fe distances are always in between those obtained for the two minima. Nevertheless, it is difficult to determine which of the two minima corresponds to the crystal structure, probably owing to the mediocre accuracy of the crystal structures and the possibility that they are partially photoreduced during crystallography and therefore may represent a mixture of oxidation states due to the photoreduction process. However, the calculations indicate that the L state is always more stable than the S state.

Moreover, we investigated whether the two local minima could also be obtained with other DFT functionals, as well as the dependence of the results on the basis set. Using seven other functionals (both pure and hybrid functionals) along with two basis sets, we optimized the cluster structures. Interestingly, while all methods could give the L local minimum, the S minimum was only obtained with some pure functionals. In particular, the S minimum was never found with the r^2^SCAN, TPSSh, B3LYP, and B3LYP*. Increasing the basis set often led to the disappearance of the S minimum, also with the pure functionals. Considering that the S minimum is less stable than the L minimum and that is found mainly with the small basis set and with the older DFT functionals, it is likely that it represents a spurious artifact, rather than a real alternative that could be observed experimentally.

Therefore, we finally compared the structures of the L local minimum optimized with the nine DFT methods and the two basis sets with the starting crystal structures. The results in Tables 6 and 7 show that r^2^SCAN/def2-SV(P) gives the best structures. Considering that this method is quite fast and it does not give the spurious S local minimum, we recommend this approach for optimizing a [4Fe–4S] cluster in proteins.

## Supplementary Information

Supplementary Information.

## Data Availability

All data generated or analysed during this study are included in this published article and its supplementary information file.
