# Supplementary material for: Two local minima for structures of [4Fe–4S] clusters obtained with density functional theory methods
Source: Sci Rep. 2023 Jul 4;13:10832. doi: 10.1038/s41598-023-37755-0 (PMC10319735; doi:10.1038/s41598-023-37755-0)
Supplement: Supplementary file 1 — Supplementary Information. [file 41598_2023_37755_MOESM1_ESM.docx]

***Supplementary Information***

Two local minima for structures of [4Fe– 4S] clusters obtained with density functional theory methods

Sonia Jafari, ^1^ Ulf Ryde, ^2^ Mehdi Irani^1*^

^1^ Department of Chemistry, University of Kurdistan, P.O. Box 66175-416, Sanandaj, Iran

^2^ Department of Theoretical Chemistry, Lund University, Chemical Centre, P.O. Box 124,
SE-221 00 Lund, Sweden

Correspondence to Mehdi Irani, E-mail: m.irani@uok.ac.ir,

Tel: +98 – 9128018046

2023-06-30


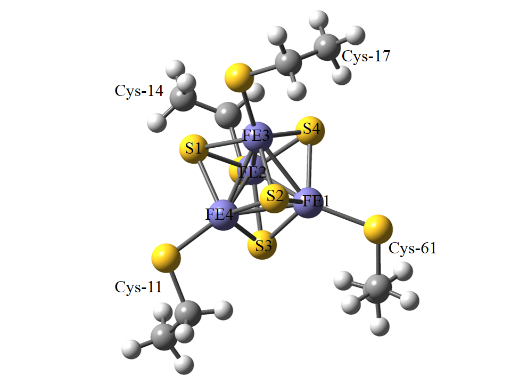

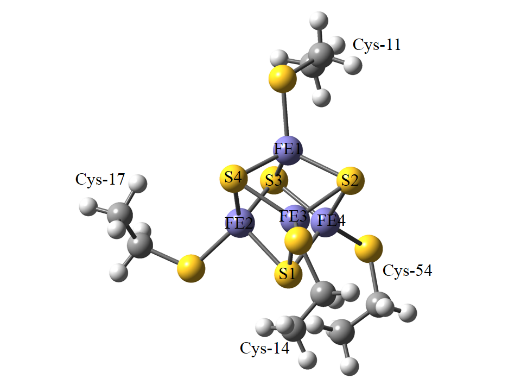

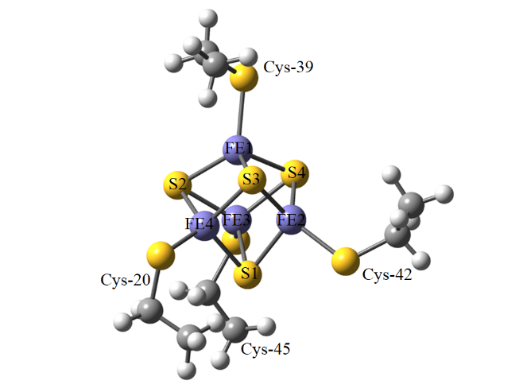


(a) (b) (c)


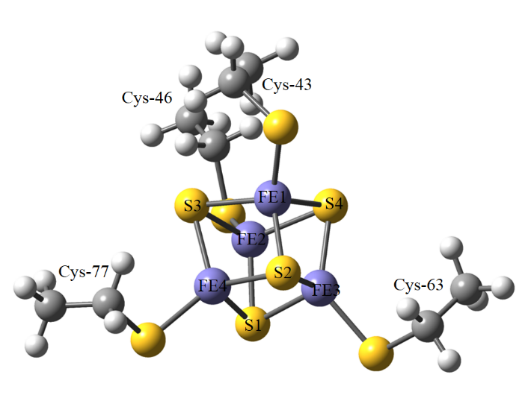

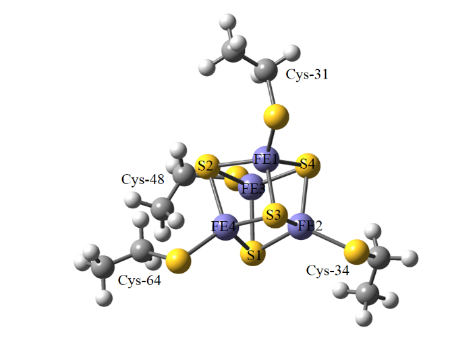


(d) (e)

Figure S1. Nomenclature of the atoms in the [4Fe-4S] cluster in the crystal structure of (a) 4Fd1, (b) 4Fd2 (c) 4Fd3 (d) Hip1, and (e) Hip2 systems.

# Details of the QM/MM Calculations

In the QM/MM calculations,^1,2^ we split the protein and solvent into two subsystems: System 1 (the QM region) was treated by QM methods. System 2 contained the remaining part of the protein and the solvent. It was kept fixed at the original coordinates (equilibrated crystal structure).

In the QM calculations, system 1 was represented by a wave function, whereas all the other atoms were represented by an array of partial point charges, one for each atom, taken from MM libraries. Thereby, the polarization of the QM system by the surroundings is included in a self-consistent manner. When there is a bond between systems 1 and 2 (a junction), the hydrogen link-atom approach was employed. In this approach, the QM system was capped with hydrogen atoms (hydrogen link atoms, HL), the positions of which are linearly related to the corresponding carbon atoms (carbon link atoms, CL) in the full system.^3,4^ All atoms were included in the point-charge model, except the CL atoms.^5^ The point charges do not necessarily sum up to an integer, because the Amber force field does not employ charge groups.^6^

The total QM/MM energy in ComQum was calculated as^4,7^

$E_{\text{QM/MM}}=E_{\text{QM1+ptch2}}^{\text{HL}}+E_{\text{MM12,}q_{\text{1}}\text{=0}}^{\text{CL}}-E_{\text{MM1,}q_{\text{1}}\text{=0}}^{\text{HL}}$ (1)

where $E_{\text{QM1+ptch2}}^{\text{HL}}$ $E_{\text{QM1+ptch23}}^{\text{HL}}$ is the QM energy of system 1 truncated by HL atoms and embedded in the set of point charges modeling of system 2 (but excluding the self-energy of the point charges). $E_{\text{MM1,}q_{\text{1}}\text{=0}}^{\text{HL}}$ is the MM energy of system 1, still truncated by HL atoms but without any electrostatic interactions. Finally, $E_{\text{MM12,}q_{\text{1}}\text{=0}}^{\text{CL}}$ is the classical energy of all atoms in the model with CL atoms and with the charges of the QM region set to zero (to avoid double-counting of the electrostatic interactions). Thus, ComQum employs a subtractive scheme with electrostatic embedding and van der Waals link-atom corrections.^8^ No cut-off is used for any of the interactions in the three energy terms in equation (1). The geometry optimizations were continued until the energy change between the two iterations was less than 2.6 J/mol (10^–6^ a.u.), and the maximum norm of the Cartesian gradients was below 10^–3^ a.u.

Table S1. Key geometrical parameters of the [4Fe-4S] cluster of 4Fd1, in its crystal structure (1IQZ PDB ID) and the calculated local minima in the Ox and Red states (at the TPSS/def2-SV(P) level of theory). The parameters include Fe-Fe, Fe-inorganic sulfur (S1, S2, S3, and S4) distances, and distances between the Fe ions and SG atoms of the cysteine residues in the crystal structure (Cys-11, -14, -17, and -61). The atom names in the first column are according to the 1IQZ PDB structure (the names are indicated in Figure S1a). The last five rows show the average of Fe-S (Ave_Fe-S_), and Fe-Fe distances (Ave_Fe-Fe_), as well as the mean absolute deviations (MADs) from the corresponding crystal structures of all distances (MAD_All_), the MAD of Fe-S (MAD_Fe-S_), and Fe-Fe (MAD_Fe-Fe_) distances. The values are all in Å.

| Atom1-Atom2 | Crystal, 1IQZ | DFT | | | |
| --- | --- | --- | --- | --- | --- |
|  |  | Ox | | Red | |
|  |  | **4Fd1-Ox-L** | **4Fd1-Ox-S** | **4Fd1-Red-L** | **4Fd1-Red-S** |
| FE1-Cys61 | 2.27 | 2.28 | 2.29 | 2.31 | 2.32 |
| FE1-S2 | 2.28 | 2.27 | 2.25 | 2.31 | 2.30 |
| FE1-S3 | 2.30 | 2.36 | 2.34 | 2.37 | 2.35 |
| FE1-S4 | 2.29 | 2.36 | 2.34 | 2.39 | 2.35 |
| FE1-FE3 | 2.73 | 2.78 | 2.63 | 2.79 | 2.65 |
| FE1-FE2 | 2.71 | 2.76 | 2.63 | 2.74 | 2.63 |
| FE1-FE4 | 2.72 | 2.77 | 2.64 | 2.77 | 2.65 |
| FE4-Cys11 | 2.30 | 2.29 | 2.30 | 2.35 | 2.36 |
| FE4-S2 | 2.28 | 2.34 | 2.31 | 2.35 | 2.33 |
| FE4-S3 | 2.27 | 2.27 | 2.24 | 2.28 | 2.25 |
| FE4-S1 | 2.29 | 2.36 | 2.33 | 2.36 | 2.34 |
| FE4-FE2 | 2.73 | 2.79 | 2.66 | 2.80 | 2.66 |
| FE4-FE3 | 2.72 | 2.76 | 2.62 | 2.73 | 2.56 |
| FE2-Cys14 | 2.27 | 2.26 | 2.27 | 2.26 | 2.27 |
| FE2-S3 | 2.29 | 2.33 | 2.31 | 2.33 | 2.32 |
| FE2-S4 | 2.31 | 2.37 | 2.35 | 2.40 | 2.37 |
| FE2-S1 | 2.28 | 2.28 | 2.26 | 2.34 | 2.31 |
| FE2-FE3 | 2.74 | 2.77 | 2.64 | 2.76 | 2.61 |
| FE3-Cys17 | 2.27 | 2.25 | 2.25 | 2.30 | 2.31 |
| FE3-S2 | 2.31 | 2.36 | 2.33 | 2.39 | 2.36 |
| FE3-S4 | 2.27 | 2.27 | 2.25 | 2.28 | 2.26 |
| FE3-S1 | 2.31 | 2.39 | 2.36 | 2.42 | 2.39 |
| Ave_Fe-S_ | 2.29 | 2.31 | 2.30 | 2.34 | 2.32 |
| Ave_Fe-Fe_ | 2.73 | 2.77 | 2.64 | 2.76 | 2.63 |
| MAD | MAD_All_ | 0.04 | 0.04 | 0.05 | 0.06 |
|  | MAD_Fe-S_ | 0.03 | 0.03 | 0.05 | 0.04 |
|  | MAD_Fe-Fe_ | 0.05 | 0.09 | 0.04 | 0.10 |

Table S2. Key geometrical parameters of the [4Fe-4S] cluster of 4Fd2, in its crystal structure (1FXR PDB ID) and the calculated local minima in the Ox and Red states (at the TPSS/def2-SV(P) level of theory). The parameters include Fe-Fe, Fe-inorganic sulfur (S1, S2, S3, and S4) distances, and distances between the Fe ions and SG atoms of the cysteine residues in the crystal structure (Cys-11, -14, -17, and -54). The atom names in the first column are according to the 1FXR PDB structure (the names are indicated in Figure S1b). The last five rows show the average of Fe-S (Ave_Fe-S_), and Fe-Fe distances (Ave_Fe-Fe_), as well as the mean absolute deviations (MADs) from the corresponding crystal structures of all distances (MAD_All_), the MAD of Fe-S (MAD_Fe-S_), and Fe-Fe (MAD_Fe-Fe_) distances. The values are all in Å.

|  |  | DFT | | | |
| --- | --- | --- | --- | --- | --- |
| Atom1-Atom2 | Crystal, 1FXR | Ox | | Red | |
|  |  | **4Fd2-Ox-L** | **4Fd2-Ox-S** | **4Fd2-Red-L** | **4Fd2-Red-S** |
| FE1-CYS-11 | 2.31 | 2.30 | 2.31 | 2.36 | 2.37 |
| FE1-S2 | 2.31 | 2.27 | 2.25 | 2.29 | 2.26 |
| FE1-S3 | 2.28 | 2.34 | 2.32 | 2.36 | 2.34 |
| FE1-S4 | 2.32 | 2.36 | 2.33 | 2.36 | 2.34 |
| FE1-FE2 | 2.73 | 2.77 | 2.67 | 2.74 | 2.60 |
| FE1-FE3 | 2.80 | 2.80 | 2.66 | 2.79 | 2.65 |
| FE1-FE4 | 2.70 | 2.75 | 2.64 | 2.75 | 2.66 |
| FE3-CYS-14 | 2.00 | 2.24 | 2.25 | 2.23 | 2.24 |
| FE3-S1 | 2.31 | 2.36 | 2.34 | 2.40 | 2.37 |
| FE3-S2 | 2.30 | 2.34 | 2.31 | 2.33 | 2.31 |
| FE3-S4 | 2.27 | 2.28 | 2.26 | 2.34 | 2.31 |
| FE3-FE2 | 2.76 | 2.76 | 2.62 | 2.76 | 2.60 |
| FE3-FE4 | 2.66 | 2.76 | 2.63 | 2.75 | 2.63 |
| FE2-CYS-17 | 2.23 | 2.26 | 2.27 | 2.32 | 2.33 |
| FE2-S1 | 2.30 | 2.25 | 2.23 | 2.25 | 2.24 |
| FE2-S3 | 2.30 | 2.35 | 2.33 | 2.37 | 2.35 |
| FE2-S4 | 2.34 | 2.38 | 2.35 | 2.40 | 2.38 |
| FE2-FE4 | 2.75 | 2.79 | 2.66 | 2.79 | 2.68 |
| FE4-CYS-54 | 2.14 | 2.30 | 2.30 | 2.34 | 2.35 |
| FE4-S1 | 2.33 | 2.36 | 2.33 | 2.38 | 2.34 |
| FE4-S2 | 2.32 | 2.37 | 2.35 | 2.37 | 2.35 |
| FE4-S3 | 2.31 | 2.29 | 2.27 | 2.34 | 2.33 |
| Ave_Fe-S_ | 2.27 | 2.32 | 2.30 | 2.34 | 2.33 |
| Ave_Fe-Fe_ | 2.73 | 2.77 | 2.65 | 2.76 | 2.64 |
| MAD | MAD_All_ | 0.05 | 0.06 | 0.06 | 0.07 |
|  | MAD_Fe-S_ | 0.06 | 0.05 | 0.08 | 0.07 |
|  | MAD_Fe-Fe_ | 0.04 | 0.09 | 0.03 | 0.10 |

Table S3. Key geometrical parameters of the [4Fe-4S] cluster of 4Fd3, in its crystal structure (5FD1 PDB ID) and the calculated local minima in the Ox and Red states (at the TPSS/def2-SV(P) level of theory). The parameters include Fe-Fe, Fe-inorganic sulfur (S1, S2, S3, and S4) distances, and distances between the Fe ions and SG atoms of the cysteine residues in the crystal structure (Cys-20, -39, -42, and -45). The atom names in the first column are according to the 5FD1 PDB structure (the names are indicated in Figure S1c). The last five rows show the average of Fe-S (Ave_Fe-S_), and Fe-Fe distances (Ave_Fe-Fe_), as well as the mean absolute deviations (MADs) from the corresponding crystal structures of all distances (MAD_All_), the MAD of Fe-S (MAD_Fe-S_), and Fe-Fe (MAD_Fe-Fe_) distances. The values are all in Å.

|  |  | DFT | | | |
| --- | --- | --- | --- | --- | --- |
| Atom1-Atom2 | Crystal, 5FD1 | Ox | | Red | |
|  |  | **4Fd3-Ox-L** | **4Fd3-Ox-S** | **4Fd3-Red-L** | **4Fd3-Red-S** |
| FE1-CYS-39 | 2.31 | 2.31 | 2.32 | 2.32 | 2.33 |
| FE1-S2 | 2.31 | 2.32 | 2.31 | 2.33 | 2.32 |
| FE1-S3 | 2.32 | 2.26 | 2.24 | 2.31 | 2.29 |
| FE1-S4 | 2.30 | 2.34 | 2.31 | 2.34 | 2.31 |
| FE1-FE2 | 2.75 | 2.80 | 2.69 | 2.82 | 2.71 |
| FE1-FE3 | 2.64 | 2.78 | 2.65 | 2.75 | 2.64 |
| FE1-FE4 | 2.75 | 2.76 | 2.65 | 2.76 | 2.67 |
| FE2-CYS-42 | 2.30 | 2.28 | 2.29 | 2.34 | 2.35 |
| FE2-S1 | 2.25 | 2.33 | 2.32 | 2.35 | 2.34 |
| FE2-S3 | 2.27 | 2.32 | 2.31 | 2.34 | 2.32 |
| FE2-S4 | 2.31 | 2.28 | 2.26 | 2.29 | 2.28 |
| FE2-FE3 | 2.70 | 2.76 | 2.63 | 2.77 | 2.63 |
| FE2-FE4 | 2.72 | 2.77 | 2.65 | 2.73 | 2.60 |
| FE3-CYS-45 | 2.29 | 2.23 | 2.24 | 2.25 | 2.27 |
| FE3-S1 | 2.28 | 2.25 | 2.23 | 2.31 | 2.28 |
| FE3-S2 | 2.27 | 2.35 | 2.32 | 2.35 | 2.32 |
| FE3-S4 | 2.29 | 2.37 | 2.34 | 2.40 | 2.36 |
| FE3-FE4 | 2.72 | 2.79 | 2.64 | 2.79 | 2.65 |
| FE4-CYS-20 | 2.31 | 2.31 | 2.31 | 2.38 | 2.38 |
| FE4-S1 | 2.28 | 2.37 | 2.34 | 2.39 | 2.36 |
| FE4-S2 | 2.31 | 2.27 | 2.25 | 2.29 | 2.27 |
| FE4-S3 | 2.29 | 2.35 | 2.34 | 2.36 | 2.35 |
| Ave_Fe-S_ | 2.29 | 2.31 | 2.30 | 2.33 | 2.32 |
| Ave_Fe-Fe_ | 2.71 | 2.78 | 2.65 | 2.77 | 2.65 |
| MAD | MAD_All_ | 0.05 | 0.05 | 0.05 | 0.05 |
|  | MAD_Fe-S_ | 0.05 | 0.04 | 0.05 | 0.04 |
|  | MAD_Fe-Fe_ | 0.06 | 0.06 | 0.06 | 0.06 |

Table S4. Key geometrical parameters of the [4Fe-4S] cluster of Hip1, in its crystal structure (1CKU PDB ID) and the calculated local minima in the Ox and Red states (at the TPSS/def2-SV(P) level of theory). The parameters include Fe-Fe, Fe-inorganic sulfur (S1, S2, S3, and S4) distances, and distances between the Fe ions and SG atoms of the cysteine residues in the crystal structure (Cys-43, -46, -63, and -77). The atom names in the first column are according to the 1CKU PDB structure (the names are indicated in Figure S1d). The last five rows show the average of Fe-S (Ave_Fe-S_), and Fe-Fe distances (Ave_Fe-Fe_), as well as the mean absolute deviations (MADs) from the corresponding crystal structures of all distances (MAD_All_), the MAD of Fe-S (MAD_Fe-S_), and Fe-Fe (MAD_Fe-Fe_) distances. The values are all in Å.

|  |  | DFT | | | |
| --- | --- | --- | --- | --- | --- |
| Atom1-Atom2 | Crystal, 1CKU | Ox | | Red | |
|  |  | **Hip1-Ox-L** | **Hip1-Ox-S** | **Hip1-Red-L** | **Hip1-Red-S** |
| FE1CYS-43 | 2.25 | 2.21 | 2.21 | 2.26 | 2.26 |
| FE1-S2 | 2.24 | 2.22 | 2.25 | 2.26 | 2.23 |
| FE1-S3 | 2.33 | 2.38 | 2.29 | 2.37 | 2.33 |
| FE1-S4 | 2.31 | 2.33 | 2.28 | 2.34 | 2.31 |
| FE1-FE2 | 2.73 | 2.94 | 2.61 | 2.79 | 2.63 |
| FE1-FE4 | 2.71 | 2.79 | 2.57 | 2.78 | 2.60 |
| FE1-FE3 | 2.70 | 2.75 | 2.60 | 2.76 | 2.63 |
| FE4-CYS-77 | 2.27 | 2.22 | 2.20 | 2.26 | 2.26 |
| FE4-S2 | 2.29 | 2.34 | 2.23 | 2.35 | 2.31 |
| FE4-S3 | 2.28 | 2.29 | 2.22 | 2.31 | 2.28 |
| FE4-S1 | 2.29 | 2.31 | 2.23 | 2.32 | 2.29 |
| FE2-FE4 | 2.71 | 2.75 | 2.57 | 2.76 | 2.59 |
| FE2-FE3 | 2.70 | 2.77 | 2.61 | 2.77 | 2.63 |
| FE2-CYS-46 | 2.30 | 2.21 | 2.21 | 2.25 | 2.26 |
| FE2-S3 | 2.30 | 2.38 | 2.31 | 2.39 | 2.36 |
| FE2-S4 | 2.29 | 2.36 | 2.30 | 2.36 | 2.33 |
| FE2-S1 | 2.23 | 2.21 | 2.25 | 2.25 | 2.23 |
| FE4-FE3 | 2.75 | 2.81 | 2.53 | 2.79 | 2.63 |
| FE3-CYS-63 | 2.27 | 2.22 | 2.23 | 2.27 | 2.28 |
| FE3-S2 | 2.32 | 2.36 | 2.30 | 2.37 | 2.35 |
| FE3-S4 | 2.25 | 2.24 | 2.21 | 2.25 | 2.23 |
| FE3-S1 | 2.31 | 2.35 | 2.28 | 2.36 | 2.34 |
| Ave_Fe-S_ | 2.28 | 2.29 | 2.25 | 2.31 | 2.29 |
| Ave_Fe-Fe_ | 2.72 | 2.80 | 2.58 | 2.78 | 2.62 |
| MAD | MAD_All_ | 0.05 | 0.07 | 0.04 | 0.04 |
|  | MAD_Fe-S_ | 0.04 | 0.04 | 0.03 | 0.02 |
|  | MAD_Fe-Fe_ | 0.08 | 0.14 | 0.06 | 0.10 |

Table S5. Key geometrical parameters of the [4Fe-4S] cluster of Hip2, in its crystal structure (2HIP PDB ID) and the calculated local minima in the Ox and Red states. The parameters include Fe-Fe, Fe-inorganic sulfur (S1, S2, S3, and S4) distances, and distances between the Fe ions and SG atoms of the cysteine residues in the crystal structure (Cys-31, -34, -48, and -64). The atom names in the first column are according to the 2HIP PDB structure (the names are indicated in Figure S1e). The last five rows show the average of Fe-S (Ave_Fe-S_), and Fe-Fe distances (Ave_Fe-Fe_), as well as the mean absolute deviations (MADs) from the corresponding crystal structures of all distances (MAD_All_), the MAD of Fe-S (MAD_Fe-S_), and Fe-Fe (MAD_Fe-Fe_) distances. The values are all in Å.

|  |  | DFT | | | |
| --- | --- | --- | --- | --- | --- |
| Atom1-Atom2 | Crystal, 2HIP | Ox | | Red | |
|  |  | Hip2-Ox-L | Hip2-Ox-S | Hip2-Red-L | Hip2-Red-S |
| FE1-CYS-31 | 2.18 | 2.22 | 2.22 | 2.27 | 2.28 |
| FE1-S2 | 2.22 | 2.22 | 2.26 | 2.26 | 2.24 |
| FE1-S3 | 2.22 | 2.38 | 2.29 | 2.36 | 2.33 |
| FE1-S4 | 2.22 | 2.34 | 2.29 | 2.35 | 2.33 |
| FE1-FE2 | 2.64 | 2.96 | 2.62 | 2.79 | 2.65 |
| FE1-FE3 | 2.66 | 2.76 | 2.63 | 2.77 | 2.65 |
| FE1-FE4 | 2.65 | 2.78 | 2.54 | 2.77 | 2.58 |
| FE2- CYS-34 | 2.42 | 2.23 | 2.23 | 2.28 | 2.29 |
| FE2-S1 | 2.22 | 2.21 | 2.25 | 2.25 | 2.23 |
| FE2-S3 | 2.22 | 2.39 | 2.32 | 2.39 | 2.37 |
| FE2-S4 | 2.22 | 2.37 | 2.31 | 2.36 | 2.34 |
| FE2-FE3 | 2.65 | 2.77 | 2.63 | 2.76 | 2.64 |
| FE2-FE4 | 2.66 | 2.76 | 2.57 | 2.77 | 2.60 |
| FE3-CYS-48 | 2.09 | 2.23 | 2.23 | 2.28 | 2.29 |
| FE3-S1 | 2.22 | 2.34 | 2.27 | 2.36 | 2.34 |
| FE3-S2 | 2.22 | 2.36 | 2.30 | 2.38 | 2.35 |
| FE3-S4 | 2.22 | 2.26 | 2.22 | 2.26 | 2.25 |
| FE3-FE4 | 2.67 | 2.80 | 2.51 | 2.78 | 2.61 |
| FE4-CYS-64 | 2.02 | 2.21 | 2.19 | 2.25 | 2.25 |
| FE4-S1 | 2.22 | 2.31 | 2.22 | 2.32 | 2.29 |
| FE4-S2 | 2.22 | 2.35 | 2.24 | 2.37 | 2.32 |
| FE4-S3 | 2.22 | 2.28 | 2.22 | 2.30 | 2.27 |
| Ave_All_ | 2.33 | 2.43 | 2.34 | 2.44 | 2.38 |
| Ave_Fe-S_ | 2.21 | 2.29 | 2.25 | 2.32 | 2.30 |
| Ave_Fe-Fe_ | 2.66 | 2.81 | 2.58 | 2.77 | 2.62 |
| MAD | MAD_All_ | 0.12 | 0.07 | 0.12 | 0.08 |
|  | MAD_Fe-S_ | 0.11 | 0.07 | 0.12 | 0.10 |
|  | MAD_Fe-Fe_ | 0.15 | 0.07 | 0.12 | 0.04 |

# Spin states of the local minima at the TPSS/ def2-TZVPD and B3LYP/def2-SV(P) levels of theory

Mulliken spin populations of the iron ions in the various oxidation states and local minima at the TPSS/ def2-TZVPD and B3LYP/def2-SV(P) levels of theory are collected in Table S6. It can be seen that enlarging the basis set from def2-SV(P) to def2-TZVPD has a small effect on the spin populations (decreasing them by –0.09 *e* on average; cf. Table S6). On the other hand, changing the functional to B3LYP increases the Fe spin populations by approximately 0.3 *e*. The S local minimum still has lower Fe spin populations, but the difference has decreased to 0.07 *e* (0.30 *e* for the oxidized HiPIP sites).

Table S6. Spin densities of the iron ions in the local minima at the TPSS/ def2-TZVPD and B3LYP/def2-SV(P) levels of theory.

|  |  | TPSS/def2-TZVPD | | | | B3LYP/def2-SV(P) | | | |
| --- | --- | --- | --- | --- | --- | --- | --- | --- | --- |
|  |  | **4Fd1-Ox-L** | **4Fd1-Ox-S** | **4Fd1-Red-L** | **4Fd1-Red-S** | **4Fd1-Ox-L** | **4Fd1-Ox-S** | **4Fd1-Red-L** | **4Fd1-Red-S** |
| 4Fd1 | Fe1 | -3.4 | -3.4 | 3.4 | 3.3 | -3.7 | -3.7 | -3.7 | -3.7 |
|  | Fe2 | 3.3 | 3.4 | 3.4 | 3.3 | 3.7 | 3.7 | 3.6 | 3.6 |
|  | Fe3 | 3.3 | 3.4 | -3.1 | -2.9 | -3.7 | -3.6 | -3.8 | -3.7 |
|  | Fe4 | -3.3 | -3.4 | -3.1 | -3.0 | 3.7 | 3.7 | 3.5 | 3.4 |
|  |  | **4Fd2-Ox-L** | **4Fd2-Ox-S** | **4Fd2-Red-L** | **4Fd2-Red-S** | **4Fd2-Ox-L** | **4Fd2-Ox-S** | **4Fd2-Red-L** | **4Fd2-Red-S** |
| 4Fd2 | Fe1 | -3.4 | -3.2 | 3.5 | 3.3 | 3.7 | 3.7 | 3.6 | 3.6 |
|  | Fe2 | 3.4 | 3.2 | -3.1 | -3.1 | -3.7 | -3.6 | -3.8 | -3.7 |
|  | Fe3 | 3.4 | 3.2 | -3.1 | -2.8 | 3.7 | 3.7 | 3.5 | 3.4 |
|  | Fe4 | -3.5 | -3.3 | 3.3 | 3.3 | -3.7 | -3.7 | -3.7 | -3.7 |
|  |  | **4Fd3-Ox-L** | **4Fd3-Ox-S** | **4Fd3-Red-L** | **4Fd3-Red-S** | **4Fd3-Ox-L** | **4Fd3-Ox-S** | **4Fd3-Red-L** | **4Fd3-Red-S** |
| 4Fd3 | Fe1 | -3.4 | -3.2 | -3.5 | -2.8 | -3.7 | -3.7 | -3.7 | -3.7 |
|  | Fe2 | -3.4 | -3.2 | -3.4 | -3.2 | 3.7 | 3.6 | 3.6 | 3.5 |
|  | Fe3 | 3.3 | 3.2 | 3.1 | 3.3 | -3.7 | -3.7 | -3.8 | -3.7 |
|  | Fe4 | 3.5 | 3.3 | 3.1 | 3.3 | 3.8 | 3.7 | 3.6 | 3.5 |
|  |  | **Hip1-Ox-L** | **Hip1-Ox-S** | **Hip1-Red-L** | **Hip1-Red-S** | **Hip1-Ox-L** | **Hip1-Ox-S** | **Hip1-Red-L** | **Hip1-Red-S** |
| Hip1 | Fe1 | 3.4 | 3.0 | 3.2 | 3.5 | -3.8 | -3.6 | -3.7 | -3.6 |
|  | Fe2 | 3.3 | 2.9 | -3.2 | -3.5 | 3.7 | 3.1 | 3.7 | 3.6 |
|  | Fe3 | -3.6 | -2.7 | -3.2 | -3.4 | -3.8 | -3.6 | -3.7 | -3.6 |
|  | Fe4 | -3.6 | -2.4 | 3.2 | 3.4 | 3.6 | 3.5 | 3.7 | 3.6 |
|  |  | **Hip2-Ox-L** | **Hip2-Ox-S** | **Hip2-Red-L** | **Hip2-Red-S** | **Hip2-Ox-L** | **Hip2-Ox-S** | **Hip2-Red-L** | **Hip2-Red-S** |
| Hip2 | Fe1 | -3.6 | 3.0 | -3.5 | -3.2 | 3.6 | 3.5 | 3.7 | 3.7 |
|  | Fe2 | 3.4 | -2.5 | -3.5 | -3.2 | 3.7 | 3.1 | 3.7 | 3.6 |
|  | Fe3 | 3.4 | 2.9 | 3.5 | 3.2 | -3.8 | -3.6 | -3.7 | -3.6 |
|  | Fe4 | -3.6 | -2.7 | 3.4 | 3.2 | -3.8 | -3.6 | -3.7 | -3.6 |

Table S7. Calculated key geometrical parameters of the L local minimum of the [4Fe-4S] cluster of 4Fd1 using the TPSS, BLYP, PBE, BP86, B97-D, TPSSH, r^2^SCAN, B3LYP, and B3LYP* functionals, and def2-SV(P) and def2-TZVPD basis sets. The first two values at the top left corner of the table are the Ave_FeS_ and Ave_FeFe_ in the corresponding crystal structure. The values are all in Å.

| 4Fd1 | | | | | | | | | | | | | | | | | | |
| --- | --- | --- | --- | --- | --- | --- | --- | --- | --- | --- | --- | --- | --- | --- | --- | --- | --- | --- |
| Ave_Fe-S_(Crystal) | 2.29 | def2-SV(P) | | | | | | | | | def2-TZVPD | | | | | | | |
| Ave_Fe-Fe_(Crystal) | 2.73 | TPSS | BLYP | PBE | BP86 | B97-D | TPSSH | r2SCAN | B3LYP | B3LYP* | TPSS | BLYP | PBE | BP86 | B97-D | TPSSH | r2SCAN | B3LYP* |
| Ave_Fe-S_ | 4Fd1-Ox-L | 2.31 | 2.32 | 2.31 | 2.31 | 2.32 | 2.32 | 2.31 | 2.34 | 2.34 | 2.29 | 2.19 | 2.28 | 2.28 | 2.30 | 2.30 | 2.29 | 2.32 |
|  | 4Fd1-Red-L | 2.30 | 2.35 | 2.33 | 2.33 | 2.35 | 2.35 | 2.33 | 2.37 | 2.37 | 2.31 | 2.31 | 2.30 | 2.29 | 2.32 | 2.33 | 2.32 | 2.36 |
| Ave_Fe-Fe_ | 4Fd1-Ox-L | 2.77 | 2.77 | 2.77 | 2.78 | 2.78 | 2.78 | 2.74 | 2.83 | 2.83 | 2.78 | 2.81 | 2.78 | 2.79 | 2.79 | 2.78 | 2.78 | 2.83 |
|  | 4Fd1-Red-L | 2.76 | 2.76 | 2.77 | 2.77 | 2.77 | 2.79 | 2.74 | 2.85 | 2.85 | 2.78 | 2.78 | 2.78 | 2.78 | 2.78 | 2.79 | 2.77 | 2.87 |
| MAD_All_ | 4Fd1-Ox-L | 0.04 | 0.04 | 0.04 | 0.04 | 0.04 | 0.04 | 0.03 | 0.07 | 0.06 | 0.04 | 0.09 | 0.04 | 0.04 | 0.04 | 0.03 | 0.04 | 0.06 |
|  | 4Fd1-Red-L | 0.05 | 0.05 | 0.05 | 0.04 | 0.06 | 0.07 | 0.05 | 0.10 | 0.10 | 0.04 | 0.04 | 0.04 | 0.04 | 0.04 | 0.05 | 0.04 | 0.09 |
| MAD_Fe-S_ | 4Fd1-Ox-L | 0.03 | 0.04 | 0.04 | 0.04 | 0.04 | 0.04 | 0.02 | 0.05 | 0.05 | 0.03 | 0.09 | 0.03 | 0.04 | 0.03 | 0.02 | 0.03 | 0.04 |
|  | 4Fd1-Red-L | 0.05 | 0.06 | 0.05 | 0.04 | 0.06 | 0.07 | 0.05 | 0.09 | 0.08 | 0.03 | 0.04 | 0.03 | 0.03 | 0.04 | 0.05 | 0.04 | 0.07 |
| MAD_Fe-Fe_ | 4Fd1-Ox-L | 0.05 | 0.05 | 0.05 | 0.05 | 0.05 | 0.05 | 0.03 | 0.10 | 0.10 | 0.06 | 0.08 | 0.06 | 0.06 | 0.06 | 0.06 | 0.05 | 0.11 |
|  | 4Fd1-Red-L | 0.04 | 0.04 | 0.04 | 0.05 | 0.04 | 0.08 | 0.04 | 0.14 | 0.13 | 0.05 | 0.05 | 0.05 | 0.06 | 0.05 | 0.07 | 0.04 | 0.15 |

Table S8. Calculated key geometrical parameters of the L local minimum of the [4Fe-4S] cluster of 4Fd2 using the TPSS, BLYP, PBE, BP86, B97-D, TPSSH, r^2^SCAN, B3LYP and B3LYP* functionals, and def2-SV(P) and def2-TZVPD basis sets. The first two values at the top left corner of the table are the Ave_FeS_ and Ave_FeFe_ in the corresponding crystal structure. The values are all in Å.

| 4Fd2 | | | | | | | | | | | | | | | | | | |
| --- | --- | --- | --- | --- | --- | --- | --- | --- | --- | --- | --- | --- | --- | --- | --- | --- | --- | --- |
| Ave_Fe-S_(Crystal) | 2.27 | def2-SV(P) | | | | | | | | | def2-TZVPD | | | | | | | |
| Ave_Fe-Fe_(Crystal) | 2.73 | TPSS | BLYP | PBE | BP86 | B97-D | TPSSH | r^2^SCAN | B3LYP | B3LYP* | TPSS | BLYP | PBE | BP86 | B97-D | TPSSH | r^2^SCAN | B3LYP* |
| Ave_Fe-S_ | 4Fd2-Ox-L | 2.32 | 2.32 | 2.31 | 2.31 | 2.33 | 2.32 | 2.31 | 2.34 | 2.34 | 2.29 | 2.30 | 2.28 | 2.28 | 2.30 | 2.30 | 2.30 | 2.33 |
|  | 4Fd2-Red-L | 2.34 | 2.34 | 2.33 | 2.33 | 2.35 | 2.36 | 2.34 | 2.38 | 2.38 | 2.31 | 2.31 | 2.30 | 2.29 | 2.32 | 2.33 | 2.32 | 2.36 |
| Ave_Fe-Fe_ | 4Fd2-Ox-L | 2.77 | 2.77 | 2.77 | 2.78 | 2.78 | 2.78 | 2.76 | 2.85 | 2.85 | 2.78 | 2.78 | 2.78 | 2.79 | 2.79 | 2.78 | 2.79 | 2.86 |
|  | 4Fd2-Red-L | 2.76 | 2.76 | 2.77 | 2.77 | 2.77 | 2.79 | 2.75 | 2.88 | 2.88 | 2.78 | 2.78 | 2.78 | 2.78 | 2.80 | 2.80 | 2.79 | 2.89 |
| MAD_All_ | 4Fd2-Ox-L | 0.05 | 0.06 | 0.05 | 0.05 | 0.06 | 0.05 | 0.04 | 0.08 | 0.08 | 0.06 | 0.06 | 0.05 | 0.05 | 0.06 | 0.05 | 0.05 | 0.07 |
|  | 4Fd2-Red-L | 0.06 | 0.06 | 0.06 | 0.06 | 0.07 | 0.08 | 0.07 | 0.12 | 0.12 | 0.05 | 0.07 | 0.05 | 0.05 | 0.06 | 0.06 | 0.05 | 0.11 |
| MAD_Fe-S_ | 4Fd2-Ox-L | 0.06 | 0.06 | 0.06 | 0.06 | 0.06 | 0.05 | 0.05 | 0.07 | 0.07 | 0.06 | 0.06 | 0.05 | 0.05 | 0.06 | 0.04 | 0.05 | 0.05 |
|  | 4Fd2-Red-L | 0.08 | 0.08 | 0.07 | 0.06 | 0.08 | 0.09 | 0.07 | 0.11 | 0.11 | 0.06 | 0.07 | 0.06 | 0.06 | 0.06 | 0.06 | 0.05 | 0.09 |
| MAD_Fe-Fe_ | 4Fd2-Ox-L | 0.04 | 0.04 | 0.04 | 0.05 | 0.04 | 0.05 | 0.03 | 0.12 | 0.11 | 0.06 | 0.06 | 0.06 | 0.06 | 0.06 | 0.05 | 0.05 | 0.13 |
|  | 4Fd2-Red-L | 0.03 | 0.03 | 0.03 | 0.04 | 0.04 | 0.07 | 0.07 | 0.15 | 0.15 | 0.04 | 0.06 | 0.05 | 0.05 | 0.07 | 0.07 | 0.06 | 0.16 |

Table S9. Calculated key geometrical parameters of the L local minimum of the [4Fe-4S] cluster of 4Fd3 using the TPSS, BLYP, PBE, BP86, B97-D, TPSSH, r^2^SCAN, B3LYP, PBE0, and B3LYP* functionals, and def2-SV(P) and def2-TZVPD basis sets. The first two values at the top left corner of the table are the Ave_FeS_ and Ave_FeFe_ in the corresponding crystal structure. The values are all in Å.

|  | 4Fd3 | | | | | | | | | | | | | | | | | | |
| --- | --- | --- | --- | --- | --- | --- | --- | --- | --- | --- | --- | --- | --- | --- | --- | --- | --- | --- | --- |
| Ave_Fe-S_ (Crystal) | 2.29 | def2-SV(P) | | | | | | | | | | def2-TZVPD | | | | | | | |
| Ave_Fe-Fe_ (Crystal) | 2.71 | TPSS | BLYP | PBE | BP86 | B97-D | TPSSH | r^2^SCAN | B3LYP | PBE0 | B3LYP* | TPSS | BLYP | PBE | BP86 | B97-D | TPSSH | r^2^SCAN | B3LYP* |
| Ave_Fe-S_ | 4Fd3-Ox-L | 2.31 | 2.32 | 2.30 | 2.30 | 2.32 | 2.32 | 2.31 | 2.34 | 2.33 | 2.34 | 2.28 | 2.29 | 2.28 | 2.27 | 2.30 | 2.30 | 2.29 | 2.32 |
|  | 4Fd3-Red-L | 2.33 | 2.34 | 2.32 | 2.32 | 2.34 | 2.35 | 2.33 | 2.37 | 2.36 | 2.37 | 2.30 | 2.31 | 2.28 | 2.29 | 2.31 | 2.32 | 2.31 | 2.35 |
| Ave_Fe-Fe_ | 4Fd3-Ox-L | 2.78 | 2.78 | 2.78 | 2.78 | 2.78 | 2.78 | 2.77 | 2.85 | 2.86 | 2.85 | 2.77 | 2.79 | 2.79 | 2.79 | 2.79 | 2.79 | 2.79 | 2.86 |
|  | 4Fd3-Red-L | 2.77 | 2.77 | 2.77 | 2.78 | 2.77 | 2.79 | 2.77 | 2.87 | 2.87 | 2.87 | 2.78 | 2.78 | 2.79 | 2.79 | 2.79 | 2.79 | 2.80 | 2.88 |
| MAD_All_ | 4Fd3-Ox-L | 0.05 | 0.06 | 0.05 | 0.05 | 0.05 | 0.05 | 0.04 | 0.07 | 0.07 | 0.07 | 0.05 | 0.06 | 0.05 | 0.06 | 0.05 | 0.05 | 0.05 | 0.07 |
|  | 4Fd3-Red-L | 0.05 | 0.06 | 0.05 | 0.05 | 0.06 | 0.07 | 0.05 | 0.10 | 0.09 | 0.10 | 0.05 | 0.05 | 0.05 | 0.05 | 0.05 | 0.06 | 0.05 | 0.09 |
| MAD_Fe-S_ | 4Fd3-Ox-L | 0.05 | 0.05 | 0.05 | 0.05 | 0.05 | 0.04 | 0.03 | 0.05 | 0.04 | 0.05 | 0.04 | 0.05 | 0.05 | 0.05 | 0.05 | 0.04 | 0.04 | 0.04 |
|  | 4Fd3-Red-L | 0.05 | 0.06 | 0.05 | 0.05 | 0.06 | 0.06 | 0.05 | 0.08 | 0.07 | 0.08 | 0.04 | 0.05 | 0.04 | 0.05 | 0.04 | 0.05 | 0.04 | 0.06 |
| MAD_Fe-Fe_ | 4Fd3-Ox-L | 0.06 | 0.06 | 0.06 | 0.07 | 0.07 | 0.07 | 0.06 | 0.14 | 0.14 | 0.14 | 0.06 | 0.08 | 0.07 | 0.08 | 0.08 | 0.07 | 0.08 | 0.15 |
|  | 4Fd3-Red-L | 0.06 | 0.06 | 0.06 | 0.06 | 0.06 | 0.08 | 0.07 | 0.16 | 0.16 | 0.15 | 0.07 | 0.07 | 0.07 | 0.07 | 0.08 | 0.08 | 0.09 | 0.17 |

Table S10. Calculated key geometrical parameters of the L local minimum of the [4Fe-4S] cluster of Hip1 using the TPSS, BLYP, PBE, BP86, B97-D, TPSSH, r^2^SCAN, B3LYP, and B3LYP* functionals, and def2-SV(P) and def2-TZVPD basis sets. The first two values at the top left corner of the table are the Ave_FeS_ and Ave_FeFe_ in the corresponding crystal structure. The values are all in Å.

| Hip1 | | | | | | | | | | | | | | | | | | |
| --- | --- | --- | --- | --- | --- | --- | --- | --- | --- | --- | --- | --- | --- | --- | --- | --- | --- | --- |
| Ave_Fe-S_(Crystal) | 2.28 | def2-SV(P) | | | | | | | | | def2-TZVPD | | | | | | | |
| Ave_Fe-Fe_(Crystal) | 2.72 | TPSS | BLYP | PBE | BP86 | B97-D | TPSSH | r^2^SCAN | B3LYP | B3LYP* | TPSS | BLYP | PBE | BP86 | B97-D | TPSSH | r^2^SCAN | B3LYP* |
| Ave_Fe-S_ | Hip1-Ox-L | 2.29 | 2.30 | 2.28 | 2.28 | 2.30 | 2.30 | 2.28 | 2.31 | 2.31 | 2.26 | 2.25 | 2.24 | 2.23 | 2.28 | 2.28 | 2.27 | 2.30 |
|  | Hip1-Red-L | 2.31 | 2.32 | 2.31 | 2.30 | 2.32 | 2.32 | 2.30 | 2.33 | 2.33 | 2.28 | 2.29 | 2.26 | 2.27 | 2.30 | 2.30 | 2.29 | 2.32 |
| Ave_Fe-Fe_ | Hip1-Ox-L | 2.80 | 2.81 | 2.80 | 2.80 | 2.82 | 2.82 | 2.78 | 2.86 | 2.86 | 2.80 | 2.80 | 2.79 | 2.80 | 2.82 | 2.81 | 2.79 | 2.86 |
|  | Hip1-Red-L | 2.78 | 2.78 | 2.78 | 2.78 | 2.78 | 2.78 | 2.72 | 2.80 | 2.80 | 2.79 | 2.79 | 2.65 | 2.79 | 2.79 | 2.78 | 2.75 | 2.81 |
| MAD_All_ | Hip1-Ox-L | 0.05 | 0.06 | 0.05 | 0.05 | 0.06 | 0.06 | 0.05 | 0.08 | 0.08 | 0.05 | 0.05 | 0.05 | 0.06 | 0.06 | 0.05 | 0.04 | 0.07 |
|  | Hip1-Red-L | 0.04 | 0.05 | 0.04 | 0.04 | 0.05 | 0.05 | 0.03 | 0.06 | 0.06 | 0.04 | 0.04 | 0.04 | 0.04 | 0.04 | 0.03 | 0.02 | 0.05 |
| MAD_Fe-S_ | Hip1-Ox-L | 0.04 | 0.04 | 0.04 | 0.04 | 0.04 | 0.04 | 0.03 | 0.05 | 0.05 | 0.04 | 0.03 | 0.05 | 0.05 | 0.04 | 0.03 | 0.03 | 0.04 |
|  | Hip1-Red-L | 0.03 | 0.04 | 0.03 | 0.03 | 0.04 | 0.04 | 0.02 | 0.05 | 0.05 | 0.02 | 0.03 | 0.03 | 0.03 | 0.03 | 0.02 | 0.02 | 0.03 |
| MAD_Fe-Fe_ | Hip1-Ox-L | 0.08 | 0.09 | 0.09 | 0.09 | 0.10 | 0.10 | 0.08 | 0.15 | 0.15 | 0.09 | 0.08 | 0.08 | 0.08 | 0.11 | 0.10 | 0.08 | 0.15 |
|  | Hip1-Red-L | 0.06 | 0.06 | 0.06 | 0.06 | 0.06 | 0.06 | 0.03 | 0.09 | 0.08 | 0.07 | 0.07 | 0.06 | 0.07 | 0.07 | 0.07 | 0.03 | 0.10 |

Table S11. Calculated key geometrical parameters of the L local minimum of the [4Fe-4S] cluster of Hip2 using the TPSS, BLYP, PBE, BP86, B97-D, TPSSH, r^2^SCAN, B3LYP, and B3LYP* functionals, and def2-SV(P) and def2-TZVPD basis sets. The first two values at the top left corner of the table are the Ave_FeS_ and Ave_FeFe_ in the corresponding crystal structure. The values are all in Å.

| Hip2 | | | | | | | | | | | | | | | | | | |
| --- | --- | --- | --- | --- | --- | --- | --- | --- | --- | --- | --- | --- | --- | --- | --- | --- | --- | --- |
| Ave_Fe-S_(Crystal) | 2.21 | def2-SV(P) | | | | | | | | | def2-TZVPD | | | | | | | |
| Ave_Fe-Fe_(Crystal) | 2.66 | TPSS | BLYP | PBE | BP86 | B97-D | TPSSH | r^2^SCAN | B3LYP | B3LYP* | TPSS | BLYP | PBE | BP86 | B97-D | TPSSH | r^2^SCAN | B3LYP* |
| Ave_Fe-S_ | Hip2-Ox-L | 2.29 | 2.30 | 2.29 | 2.28 | 2.31 | 2.30 | 2.29 | 2.32 | 2.32 | 2.27 | 2.26 | 2.24 | 2.24 | 2.29 | 2.28 | 2.27 | 2.30 |
|  | Hip2-Red-L | 2.32 | 2.32 | 2.31 | 2.31 | 2.33 | 2.32 | 2.31 | 2.34 | 2.34 | 2.29 | 2.30 | 2.28 | 2.28 | 2.30 | 2.30 | 2.29 | 2.32 |
| Ave_Fe-Fe_ | Hip2-Ox-L | 2.81 | 2.82 | 2.81 | 2.81 | 2.82 | 2.82 | 2.79 | 2.87 | 2.86 | 2.81 | 2.80 | 2.79 | 2.80 | 2.83 | 2.82 | 2.80 | 2.87 |
|  | Hip2-Red-L | 2.77 | 2.77 | 2.77 | 2.78 | 2.78 | 2.78 | 2.73 | 2.81 | 2.81 | 2.78 | 2.79 | 2.79 | 2.79 | 2.79 | 2.78 | 2.75 | 2.82 |
| MAD_All_ | Hip2-Ox-L | 0.12 | 0.13 | 0.12 | 0.12 | 0.13 | 0.13 | 0.11 | 0.15 | 0.15 | 0.11 | 0.09 | 0.09 | 0.09 | 0.12 | 0.12 | 0.11 | 0.14 |
|  | Hip2-Red-L | 0.12 | 0.13 | 0.12 | 0.12 | 0.13 | 0.13 | 0.10 | 0.15 | 0.14 | 0.11 | 0.11 | 0.10 | 0.11 | 0.11 | 0.11 | 0.10 | 0.14 |
| MAD_Fe-S_ | Hip2-Ox-L | 0.11 | 0.12 | 0.11 | 0.10 | 0.12 | 0.12 | 0.10 | 0.13 | 0.13 | 0.09 | 0.08 | 0.07 | 0.07 | 0.10 | 0.10 | 0.09 | 0.12 |
|  | Hip2-Red-L | 0.12 | 0.13 | 0.12 | 0.12 | 0.13 | 0.13 | 0.11 | 0.14 | 0.14 | 0.10 | 0.11 | 0.10 | 0.09 | 0.11 | 0.11 | 0.10 | 0.13 |
| MAD_Fe-Fe_ | Hip2-Ox-L | 0.15 | 0.16 | 0.15 | 0.15 | 0.17 | 0.17 | 0.13 | 0.21 | 0.21 | 0.15 | 0.14 | 0.14 | 0.14 | 0.17 | 0.16 | 0.14 | 0.21 |
|  | Hip2-Red-L | 0.12 | 0.12 | 0.12 | 0.12 | 0.12 | 0.12 | 0.07 | 0.16 | 0.15 | 0.13 | 0.13 | 0.13 | 0.13 | 0.13 | 0.13 | 0.10 | 0.17 |

Table S12. Spin densities of the iron ions in various BS states of the two oxidation states of 4Fd3 at the PBE/def2-SV(P) level of theory.

|  | 4Fd3-Ox-BS1 | 4Fd3-Ox-BS2 | 4Fd3-Ox-BS3 | 4Fd3-Red-BS1 | 4Fd3-Red-BS2 | 4Fd3-Red-BS3 | 4Fd3-Red-BS4 | 4Fd3-Red-BS5 | 4Fd3-Red-BS6 |
| --- | --- | --- | --- | --- | --- | --- | --- | --- | --- |
| Fe1 | -3.37 | 3.35 | 3.34 | 3.04 | 2.93 | 3.03 | -3.42 | -3.42 | -3.37 |
| Fe2 | 3.33 | -3.31 | 3.27 | -3.46 | -3.42 | 3.21 | 3.12 | 3.09 | -3.44 |
| Fe3 | -3.35 | -3.34 | -3.25 | 3.30 | -3.46 | -3.45 | -3.49 | 3.27 | 3.16 |
| Fe4 | 3.41 | 3.37 | -3.33 | -3.47 | 3.24 | -3.42 | 3.16 | -3.48 | 3.01 |

Table S13. Calculated key geometrical parameters of the [4Fe-4S] cluster of 4Fd3 in the various BS states at the PBE/def2-SV(P) level of theory. All values are in Å.

|  | Crystal | 4Fd3-Ox-BS1 | 4Fd3-Ox-BS2 | 4Fd3-Ox-BS3 | 4Fd3-Red-BS1 | 4Fd3-Red-BS2 | 4Fd3-Red-BS3 | 4Fd3-Red-BS4 | 4Fd3-Red-BS5 | 4Fd3-Red-BS6 |
| --- | --- | --- | --- | --- | --- | --- | --- | --- | --- | --- |
| Ave_Fe-S_ | 2.29 | 2.30 | 2.30 | 2.30 | 2.32 | 2.32 | 2.32 | 2.32 | 2.33 | 2.32 |
| Ave_Fe-Fe_ | 2.71 | 2.78 | 2.78 | 2.78 | 2.77 | 2.77 | 2.78 | 2.77 | 2.77 | 2.78 |
| MAD_All_ |  | 0.05 | 0.05 | 0.05 | 0.05 | 0.05 | 0.05 | 0.05 | 0.04 | 0.05 |
| MAD_Fe-S_ |  | 0.05 | 0.04 | 0.04 | 0.05 | 0.04 | 0.04 | 0.05 | 0.04 | 0.04 |
| MAD_Fe-Fe_ |  | 0.06 | 0.07 | 0.07 | 0.06 | 0.06 | 0.06 | 0.06 | 0.06 | 0.06 |

Table S14. The energy difference between the various BS states of the two oxidation states of 4Fd3 at the PBE/def2-SV(P) level of theory. The three possible BS states of Ox were compared to each other and the six possible BS states of Red were compared to each other. All values are in kJ/mol.

| 4Fd3-Ox | | | 4Fd3-Red | | | | | |
| --- | --- | --- | --- | --- | --- | --- | --- | --- |
| BS1 | BS2 | BS3 | BS1 | BS2 | BS3 | BS4 | BS5 | BS6 |
| 0.0 | 7.2 | 4.0 | 0.0 | 5.4 | 6.1 | 7.1 | 17.4 | 22.5 |

**Cartesian coordinates of optimized QM/MM structures in the energetically most favorable BS states, obtained with the TPSS/def2-SV(P) approach.**

4Fd1-Ox-S

H -2.58300000 7.37400000 8.44600000

C -2.06400000 7.99200000 7.61000000

H -1.03200000 7.59200000 7.58800000

C -2.75800000 7.74000000 6.26600000

H -3.83300000 7.99500000 6.33900000

H -2.67600000 6.68500000 5.94200000

S -2.10700000 8.80400000 4.88000000

H -2.07500000 9.06300000 7.91000000

H 1.58000000 12.71700000 4.03800000

C 2.61900000 13.13100000 3.83600000

H 2.56300000 14.18400000 4.17100000

C 3.60200000 12.39400000 4.74700000

H 3.23100000 12.29500000 5.78600000

H 4.50100000 13.03700000 4.80000000

S 4.20500000 10.73100000 4.17100000

H 2.94200000 13.11500000 2.77100000

H 4.15200000 7.69500000 -0.48100000

C 3.92900000 6.67100000 -0.94700000

H 4.86300000 6.08400000 -0.84600000

C 2.82300000 6.04200000 -0.09300000

H 3.21500000 5.73300000 0.89100000

H 2.42800000 5.14700000 -0.61100000

S 1.38300000 7.18500000 0.20700000

H 3.67300000 6.74400000 -2.02100000

H 6.77600000 4.78200000 4.94900000

C 5.85200000 5.30600000 5.41500000

H 5.68600000 6.18300000 4.76100000

C 4.63400000 4.38200000 5.33700000

H 4.83100000 3.43500000 5.87000000

H 4.35500000 4.15000000 4.29200000

S 3.17000000 5.12000000 6.20200000

H 6.11600000 5.58200000 6.46300000

Fe 2.21200000 6.69900000 4.84800000

S 0.32800000 6.03100000 3.81800000

Fe 0.08500000 8.21700000 4.52700000

S 1.59000000 8.53200000 6.16100000

Fe 2.52000000 9.21700000 4.16100000

S 3.63400000 7.42100000 3.14200000

Fe 1.50000000 7.50100000 2.43600000

S 0.74100000 9.69500000 2.85200000

4Fd1-Ox-L

H -2.58800 7.37500 8.44600

C -2.07700 7.99400 7.60700

H -1.04900 7.58600 7.56000

C -2.81000 7.75800 6.28100

H -3.88000 8.02100 6.38800

H -2.74500 6.70600 5.94400

S -2.18400 8.83100 4.89300

H -2.07900 9.06300 7.91000

H 1.58000 12.72000 4.03800

C 2.61500 13.14300 3.83300

H 2.54800 14.20000 4.15300

C 3.61100 12.43500 4.75100

H 3.24100 12.33000 5.78900

H 4.49900 13.09300 4.80300

S 4.24500 10.78400 4.17200

H 2.94000 13.12100 2.76900

H 4.15400 7.69500 -0.48200

C 3.93600 6.67100 -0.95000

H 4.87400 6.09000 -0.85100

C 2.83100 6.02500 -0.11300

H 3.20800 5.73600 0.88300

H 2.46800 5.11700 -0.63100

S 1.35800 7.13800 0.13000

H 3.67700 6.74500 -2.02200

H 6.77700 4.78000 4.95000

C 5.85200 5.29800 5.41900

H 5.66400 6.16800 4.76300

C 4.65900 4.34000 5.35500

H 4.88900 3.40400 5.89400

H 4.38000 4.09100 4.31400

S 3.18100 5.03800 6.22200

H 6.11600 5.57900 6.46400

Fe 2.23800 6.63400 4.88700

S 0.32600 6.04400 3.81400

Fe 0.00200 8.23600 4.55400

S 1.58900 8.52500 6.14800

Fe 2.56400 9.27700 4.17000

S 3.61600 7.42500 3.13700

Fe 1.48400 7.48500 2.34800

S 0.74300 9.69500 2.86000

4Fd1-Red-S

H -2.58700000 7.37400000 8.44300000

C -2.07000000 7.99600000 7.59500000

H -1.03800000 7.59600000 7.55800000

C -2.78200000 7.74600000 6.25700000

H -3.86700000 7.95800000 6.36300000

H -2.66900000 6.69500000 5.92600000

S -2.17000000 8.84300000 4.88400000

H -2.07800000 9.06400000 7.90200000

H 1.58300000 12.72000000 4.04400000

C 2.62100000 13.13600000 3.84200000

H 2.56300000 14.19200000 4.17500000

C 3.61300000 12.39700000 4.74900000

H 3.22500000 12.28400000 5.78200000

H 4.49800000 13.06200000 4.82300000

S 4.22500000 10.74600000 4.15000000

H 2.94300000 13.11700000 2.77400000

H 4.15300000 7.69500000 -0.47900000

C 3.92700000 6.67100000 -0.94200000

H 4.85800000 6.07500000 -0.83800000

C 2.80700000 6.07000000 -0.08400000

H 3.19700000 5.78200000 0.90800000

H 2.41700000 5.16400000 -0.58900000

S 1.38800000 7.23800000 0.18900000

H 3.67200000 6.74300000 -2.01800000

H 6.77500000 4.78000000 4.94900000

C 5.84000000 5.30200000 5.42100000

H 5.65900000 6.17300000 4.76300000

C 4.63200000 4.36400000 5.34800000

H 4.85800000 3.40800000 5.85600000

H 4.35300000 4.15100000 4.29700000

S 3.17200000 5.08600000 6.22400000

H 6.11000000 5.58000000 6.46500000

Fe 0.08000000 8.25400000 4.50400000

S 0.28900000 6.02900000 3.84500000

Fe 2.21000000 6.72600000 4.89000000

S 1.56500000 8.58400000 6.16700000

Fe 1.47500000 7.53700000 2.47500000

S 3.63200000 7.39400000 3.13900000

Fe 2.53300000 9.22800000 4.16400000

S 0.71400000 9.76900000 2.84400000

4Fd1-Red-L

H -2.59000 7.37500 8.44400

C -2.08000 7.99800 7.59600

H -1.05200 7.59300 7.53700

C -2.83000 7.75700 6.27800

H -3.91200 7.96700 6.41700

H -2.72600 6.70900 5.93400

S -2.25600 8.86800 4.90200

H -2.08100 9.06500 7.90400

H 1.58200 12.72300 4.04300

C 2.61800 13.14800 3.83800

H 2.54800 14.20800 4.15500

C 3.62500 12.44200 4.75200

H 3.24400 12.34000 5.78900

H 4.50200 13.12000 4.81000

S 4.25600 10.79100 4.17300

H 2.94200 13.12200 2.77200

H 4.15500 7.69500 -0.48200

C 3.93900 6.67100 -0.94800

H 4.87600 6.08600 -0.84400

C 2.82200 6.04700 -0.10700

H 3.19600 5.78600 0.90000

H 2.47500 5.12200 -0.60700

S 1.36000 7.17200 0.10200

H 3.67800 6.74500 -2.02100

H 6.77500 4.77900 4.94900

C 5.83900 5.29600 5.42100

H 5.63800 6.15900 4.75900

C 4.66100 4.32200 5.35400

H 4.91500 3.38400 5.88200

H 4.40100 4.08100 4.30400

S 3.16400 4.99900 6.19800

H 6.10900 5.57800 6.46500

Fe -0.01100 8.27300 4.53800

S 0.27300 6.05100 3.82900

Fe 2.21500 6.67100 4.92100

S 1.55900 8.57600 6.16100

Fe 1.46900 7.51500 2.37300

S 3.61700 7.40700 3.12800

Fe 2.57100 9.28700 4.18400

S 0.71100 9.76200 2.84900

4Fd2-Ox-L

H 2.52800000 0.31100000 12.19700000

C 2.08900000 1.12600000 11.48200000

H 2.96500000 1.44500000 10.88400000

C 1.05500000 0.45900000 10.55500000

H 0.27500000 -0.05100000 11.15400000

H 1.53000000 -0.29200000 9.89400000

S 0.11100000 1.64900000 9.47600000

H 1.65800000 1.99100000 12.04200000

H 0.37700000 7.37800000 8.92200000

C 0.64200000 8.34800000 8.35300000

H 0.26000000 9.13800000 9.02700000

C 2.16800000 8.47000000 8.30700000

H 2.62200000 8.39600000 9.31800000

H 2.38800000 9.49000000 7.93600000

S 3.08400000 7.36700000 7.15400000

H 0.17300000 8.46700000 7.33500000

H 2.14400000 5.40300000 2.00300000

C 2.13000000 4.51300000 1.26300000

H 3.02400000 4.64900000 0.62200000

C 2.27300000 3.24500000 2.12800000

H 3.24000000 3.20900000 2.66200000

H 2.20700000 2.35900000 1.47000000

S 0.90900000 3.07500000 3.39100000

H 1.21500000 4.47400000 0.61700000

H 8.07500000 4.84800000 4.84600000

C 7.51800000 4.57200000 5.82800000

H 6.54000000 5.06900000 5.70000000

C 7.29800000 3.05800000 5.83000000

H 8.26000000 2.51500000 5.84700000

H 6.72400000 2.72500000 4.94500000

S 6.39800000 2.53000000 7.35600000

H 8.10200000 4.93100000 6.72500000

Fe 1.65800000 2.67700000 8.11700000

Fe 2.53800000 5.19600000 7.26400000

Fe 1.91000000 3.25100000 5.41400000

Fe 4.16700000 2.97400000 7.03300000

S 3.74000000 4.55900000 5.33500000

S 3.32900000 3.90500000 9.04600000

S 2.67200000 1.31700000 6.50900000

S 0.45300000 4.33300000 6.94800000

4Fd2-Ox-S

H 2.53000 0.31200 12.19600

C 2.09500 1.12900 11.48000

H 2.97700 1.45500 10.89600

C 1.07500 0.47300 10.53100

H 0.27800 -0.03300 11.11100

H 1.55800 -0.28200 9.87800

S 0.15900 1.67000 9.43300

H 1.66000 1.99200 12.04100

H 0.38000 7.37500 8.91900

C 0.65300 8.33900 8.34600

H 0.29400 9.13800 9.02400

C 2.18300 8.41800 8.28300

H 2.64500 8.34000 9.28900

H 2.43000 9.42800 7.90100

S 3.05800 7.28200 7.12400

H 0.17800 8.46400 7.33100

H 2.14400 5.40200 2.00500

C 2.12700 4.50800 1.27000

H 3.02500 4.62900 0.63100

C 2.25300 3.25300 2.15900

H 3.23100 3.20200 2.67300

H 2.14900 2.35400 1.52400

S 0.91400 3.15700 3.45900

H 1.21400 4.47100 0.62000

H 8.07500 4.84800 4.84600

C 7.51400 4.57700 5.82700

H 6.54400 5.08800 5.69600

C 7.26400 3.06700 5.83300

H 8.21600 2.50500 5.85800

H 6.68900 2.74300 4.94700

S 6.34500 2.56800 7.35800

H 8.10100 4.93200 6.72500

Fe 1.70100 2.70400 8.06300

Fe 2.55100 5.09700 7.26300

Fe 1.95700 3.27300 5.47200

Fe 4.10800 2.99600 7.01900

S 3.76700 4.56800 5.33500

S 3.34100 3.88100 9.05700

S 2.68300 1.30600 6.48900

S 0.45400 4.32600 6.94400

4Fd2-Red-L

H 2.52700000 0.30800000 12.19600000

C 2.07800000 1.12300000 11.47600000

H 2.94000000 1.44900000 10.86400000

C 1.03800000 0.43300000 10.56900000

H 0.30000000 -0.12000000 11.18700000

H 1.53300000 -0.29600000 9.89400000

S 0.04500000 1.60500000 9.52300000

H 1.65200000 1.99000000 12.03700000

H 0.37700000 7.38100000 8.92400000

C 0.64000000 8.35400000 8.36000000

H 0.25300000 9.14400000 9.03800000

C 2.16800000 8.47300000 8.30600000

H 2.61500000 8.39900000 9.32400000

H 2.38500000 9.50000000 7.94300000

S 3.05100000 7.34900000 7.14100000

H 0.17200000 8.46900000 7.33800000

H 2.14500000 5.40400000 2.00400000

C 2.13200000 4.51300000 1.26700000

H 3.03400000 4.64200000 0.62900000

C 2.25200000 3.25800000 2.15600000

H 3.20700000 3.24000000 2.71400000

H 2.21100000 2.36400000 1.50500000

S 0.86400000 3.12400000 3.39100000

H 1.21700000 4.47400000 0.61900000

H 8.07300000 4.84600000 4.84600000

C 7.51200000 4.56100000 5.83700000

H 6.52800000 5.04600000 5.70800000

C 7.30600000 3.04500000 5.83500000

H 8.27900000 2.51600000 5.82200000

H 6.72000000 2.72600000 4.95300000

S 6.42300000 2.50600000 7.36300000

H 8.09800000 4.92500000 6.72900000

Fe 4.15000000 2.97000000 7.07900000

Fe 1.89700000 3.26200000 5.46100000

Fe 2.54500000 5.18700000 7.32600000

Fe 1.62000000 2.67500000 8.12600000

S 3.74400000 4.54800000 5.34900000

S 3.30100000 3.88000000 9.10100000

S 2.63500000 1.27500000 6.52400000

S 0.39200000 4.34600000 6.99000000

4Fd2-Red-S

H 2.52600 0.30900 12.19400

C 2.07700 1.12600 11.47100

H 2.94600 1.45200 10.86700

C 1.04100 0.45200 10.54700

H 0.27900 -0.08100 11.15500

H 1.53200 -0.29100 9.88600

S 0.08700 1.63900 9.47700

H 1.65100 1.99100 12.03500

H 0.37900 7.37900 8.92300

C 0.64900 8.34700 8.35500

H 0.28200 9.14500 9.03500

C 2.18100 8.42500 8.28700

H 2.63500 8.34500 9.30100

H 2.42400 9.44300 7.91700

S 3.02100 7.27400 7.11400

H 0.17600 8.46700 7.33600

H 2.14400 5.40200 2.00600

C 2.12600 4.50500 1.27700

H 3.03500 4.61000 0.64600

C 2.21700 3.26700 2.19800

H 3.18300 3.22900 2.73700

H 2.13100 2.35700 1.57400

S 0.85300 3.22600 3.46900

H 1.21400 4.47000 0.62300

H 8.07300 4.84700 4.84600

C 7.50900 4.56600 5.83700

H 6.53100 5.06200 5.70500

C 7.28000 3.05400 5.84700

H 8.24400 2.50900 5.84100

H 6.69000 2.73500 4.96800

S 6.38700 2.55200 7.38300

H 8.09800 4.92800 6.72900

Fe 4.10300 3.00400 7.06000

Fe 1.91500 3.28800 5.53700

Fe 2.53400 5.09700 7.30400

Fe 1.65400 2.71300 8.06200

S 3.75600 4.55500 5.34300

S 3.30400 3.86500 9.10000

S 2.64900 1.27600 6.50200

S 0.37300 4.35400 7.00000

4Fd3-Ox-L

H 1.33300000 10.15300000 9.12100000

C 1.74000000 10.66600000 8.17100000

H 2.71800000 10.17000000 8.03900000

C 0.87600000 10.35500000 6.95200000

H -0.09900000 10.87900000 6.98600000

H 0.67500000 9.27800000 6.84900000

S 1.73800000 10.97100000 5.42800000

H 1.82200000 11.76300000 8.32800000

H 4.90100000 11.49800000 -0.45300000

C 5.42800000 10.90900000 0.36600000

H 4.82000000 11.07500000 1.27600000

C 5.38600000 9.42800000 -0.02000000

H 5.83400000 9.26500000 -1.02100000

H 4.34300000 9.06300000 -0.06600000

S 6.32600000 8.33600000 1.14600000

H 6.44900000 11.32500000 0.48900000

H 9.57600000 9.34800000 5.58300000

C 9.84000000 9.36800000 6.69400000

H 10.91400000 9.65700000 6.71900000

C 9.03100000 10.46000000 7.38200000

H 9.18300000 11.44800000 6.89900000

H 9.44300000 10.53800000 8.40800000

S 7.22600000 10.16100000 7.62200000

H 9.73500000 8.36400000 7.18300000

H 4.55000000 5.16100000 8.85200000

C 3.63600000 4.52000000 8.62900000

H 2.84800000 4.76600000 9.35900000

C 3.18200000 4.92300000 7.21600000

H 2.74900000 5.93900000 7.18200000

H 2.41100000 4.21500000 6.86700000

S 4.59700000 4.85400000 5.98400000

H 3.84300000 3.44200000 8.69500000

Fe 5.33300000 8.66200000 3.20900000

Fe 6.12400000 9.28400000 5.82600000

Fe 4.67400000 6.98600000 5.32100000

Fe 3.47100000 9.49000000 5.06500000

S 4.30400000 8.42900000 7.01000000

S 3.23900000 7.74300000 3.62700000

S 5.28600000 10.70000000 4.18400000

S 6.85700000 7.52700000 4.57200000

4Fd3-Ox-S

H 1.33500 10.15300 9.12100

C 1.74700 10.66500 8.17400

H 2.73000 10.17500 8.06000

C 0.91500 10.34300 6.93800

H -0.06000 10.86600 6.94100

H 0.71900 9.26500 6.84300

S 1.82000 10.94500 5.43400

H 1.82500 11.76300 8.32900

H 4.90000 11.49800 -0.45100

C 5.42600 10.91000 0.37300

H 4.82000 11.09100 1.28200

C 5.37500 9.42200 0.01300

H 5.82900 9.23900 -0.98200

H 4.33000 9.06600 -0.03400

S 6.29900 8.33400 1.19900

H 6.44900 11.32500 0.49100

H 9.57200 9.34600 5.58300

C 9.82900 9.36900 6.69600

H 10.89600 9.67800 6.72800

C 8.98500 10.44400 7.37200

H 9.11700 11.43400 6.88700

H 9.38400 10.53600 8.40200

S 7.18400 10.09800 7.59500

H 9.73000 8.36500 7.18400

H 4.55000 5.16200 8.85000

C 3.63800 4.52200 8.62100

H 2.84600 4.76900 9.34700

C 3.19300 4.93700 7.20600

H 2.73800 5.94300 7.18500

H 2.44100 4.21800 6.83700

S 4.61600 4.92700 5.98000

H 3.84300 3.44300 8.69000

Fe 5.31800 8.64100 3.27800

Fe 6.07700 9.23600 5.78900

Fe 4.67200 7.06600 5.30700

Fe 3.53100 9.43700 5.07000

S 4.29300 8.42400 7.03200

S 3.22400 7.73400 3.62700

S 5.29200 10.69300 4.18300

S 6.85400 7.49300 4.57400

4Fd3-Red-L

H 1.33300000 10.15200000 9.11900000

C 1.74300000 10.66700000 8.16000000

H 2.71900000 10.16500000 8.02500000

C 0.88200000 10.36300000 6.93600000

H -0.10800000 10.86100000 6.99000000

H 0.71000000 9.28200000 6.82400000

S 1.74300000 11.01600000 5.42900000

H 1.82400000 11.76400000 8.32200000

H 4.90000000 11.49600000 -0.45600000

C 5.43300000 10.89500000 0.36000000

H 4.82900000 11.03600000 1.27600000

C 5.39900000 9.42100000 -0.06100000

H 5.83200000 9.29200000 -1.07800000

H 4.35300000 9.06200000 -0.10600000

S 6.34200000 8.32900000 1.10000000

H 6.45100000 11.31800000 0.48700000

H 9.58500000 9.35300000 5.58200000

C 9.85900000 9.37300000 6.69100000

H 10.94100000 9.64200000 6.70600000

C 9.08500000 10.48600000 7.38800000

H 9.27500000 11.46500000 6.89500000

H 9.52100000 10.55600000 8.40600000

S 7.27700000 10.23900000 7.63800000

H 9.74100000 8.36700000 7.18100000

H 4.55200000 5.16100000 8.84700000

C 3.64300000 4.52100000 8.61100000

H 2.83000000 4.78900000 9.30800000

C 3.27000000 4.90600000 7.16400000

H 2.83800000 5.92200000 7.10400000

H 2.51400000 4.19400000 6.78800000

S 4.75900000 4.85600000 6.02100000

H 3.84500000 3.44300000 8.68800000

Fe 6.14500000 9.31700000 5.80600000

Fe 5.37300000 8.64400000 3.18100000

Fe 3.52800000 9.48300000 5.05600000

Fe 4.74700000 6.98800000 5.28900000

S 4.34800000 8.44200000 7.03900000

S 3.27800000 7.72900000 3.60800000

S 5.31300000 10.74100000 4.15200000

S 6.94900000 7.58400000 4.54700000

4Fd3-Red-S

H 1.33500 10.15200 9.12000

C 1.74900 10.66700 8.16300

H 2.72900 10.16900 8.04400

C 0.91700 10.35400 6.92400

H -0.07300 10.85400 6.94800

H 0.74700 9.27200 6.82000

S 1.82300 10.98800 5.43600

H 1.82600 11.76400 8.32300

H 4.90000 11.49500 -0.45400

C 5.43100 10.89500 0.36600

H 4.82800 11.04900 1.28100

C 5.39200 9.41500 -0.03200

H 5.82900 9.26700 -1.04500

H 4.34400 9.06200 -0.07700

S 6.32500 8.32900 1.14500

H 6.45100 11.31800 0.48900

H 9.58100 9.35300 5.58200

C 9.84900 9.37500 6.69100

H 10.92700 9.66000 6.71300

C 9.04300 10.47600 7.37500

H 9.21700 11.45700 6.87800

H 9.47000 10.56400 8.39600

S 7.23800 10.18400 7.61200

H 9.73700 8.36800 7.18100

H 4.55100 5.16200 8.84600

C 3.64200 4.52400 8.60500

H 2.82800 4.79100 9.30000

C 3.27500 4.92100 7.15800

H 2.82300 5.92800 7.10900

H 2.53500 4.20000 6.76600

S 4.76900 4.91800 6.02100

H 3.84400 3.44400 8.68500

Fe 6.09200 9.27600 5.77500

Fe 5.36600 8.62500 3.24900

Fe 3.59500 9.43700 5.07600

Fe 4.75400 7.06500 5.29400

S 4.34300 8.43000 7.07200

S 3.26900 7.71800 3.63100

S 5.31900 10.73400 4.14700

S 6.94500 7.55400 4.55700

Hip1-Ox-L

H -2.23500000 -2.96100000 -0.92400000

C -2.40700000 -3.15800000 0.18400000

H -1.42200000 -3.47100000 0.57300000

C -2.86200000 -1.87100000 0.85500000

H -3.01200000 -2.08600000 1.92800000

H -3.81200000 -1.49200000 0.44200000

S -1.68500000 -0.42900000 0.74400000

H -3.13500000 -3.99100000 0.33800000

H -1.35400000 -7.56500000 0.86500000

C -0.41300000 -7.49200000 1.48800000

H 0.35900000 -7.99500000 0.87800000

C -0.07700000 -6.00200000 1.61300000

H -0.80100000 -5.47400000 2.25900000

H -0.05400000 -5.49100000 0.63500000

S 1.61600000 -5.80800000 2.37200000

H -0.54000000 -7.97500000 2.48400000

H -2.22100000 -1.10900000 8.45000000

C -1.21200000 -1.52800000 8.11200000

H -1.43000000 -2.54300000 7.73600000

C -0.63200000 -0.68500000 6.97400000

H -0.55000000 0.38100000 7.25400000

H -1.23900000 -0.77100000 6.05900000

S 1.08900000 -1.27900000 6.57500000

H -0.56000000 -1.56300000 9.00100000

H 3.63100000 0.15600000 -1.02600000

C 4.63200000 0.53200000 -0.68400000

H 4.86500000 1.38700000 -1.35000000

C 4.58100000 1.02900000 0.76300000

H 3.64700000 1.56700000 1.00900000

H 5.40700000 1.74700000 0.91600000

S 4.84600000 -0.25800000 2.07300000

H 5.37200000 -0.27100000 -0.87100000

Fe 0.06800000 -1.19500000 1.85500000

S 1.24300000 0.33100000 2.94900000

Fe 2.83700000 -1.19600000 2.17200000

S 1.69500000 -2.35000000 0.56400000

Fe 1.58800000 -3.60800000 2.58200000

S -0.47000000 -2.82300000 3.43300000

Fe 1.11600000 -1.54600000 4.37100000

S 3.11100000 -2.69700000 3.90300000

Hip1-Ox-S

H -2.23500 -2.96300 -0.92300

C -2.40700 -3.16500 0.18500

H -1.42200 -3.49100 0.56600

C -2.83600 -1.88700 0.88500

H -3.01200 -2.13300 1.94800

H -3.76900 -1.46800 0.46600

S -1.60500 -0.50000 0.84100

H -3.13500 -3.99500 0.33800

H -1.35400 -7.55900 0.86600

C -0.41500 -7.47100 1.49300

H 0.37000 -7.94700 0.87800

C -0.14400 -5.96600 1.62300

H -0.90200 -5.47500 2.25600

H -0.14100 -5.46200 0.64100

S 1.51200 -5.65400 2.40100

H -0.53900 -7.96500 2.48600

H -2.21800 -1.10800 8.44400

C -1.20800 -1.53100 8.10200

H -1.43500 -2.55200 7.74800

C -0.64600 -0.71400 6.93200

H -0.57900 0.36200 7.17600

H -1.26300 -0.84200 6.02900

S 1.08200 -1.27200 6.50300

H -0.55500 -1.56200 8.99400

H 3.62900 0.15300 -1.01600

C 4.62200 0.52000 -0.64700

H 4.86200 1.39700 -1.28300

C 4.54200 0.98500 0.81000

H 3.62500 1.56100 1.03000

H 5.39500 1.66100 1.00400

S 4.70800 -0.31100 2.12900

H 5.36800 -0.27600 -0.85200

Fe 0.17200 -1.29300 1.89600

S 1.26300 0.35900 2.96200

Fe 2.70400 -1.21300 2.29900

S 1.77000 -2.28100 0.58600

Fe 1.47200 -3.45100 2.55900

S -0.49200 -2.74500 3.52300

Fe 1.17400 -1.51500 4.29000

S 3.09000 -2.68700 3.92100

Hip1-Red-S

H -2.23500000 -2.96300000 -0.92600000

C -2.40500000 -3.16400000 0.18500000

H -1.41700000 -3.48700000 0.56000000

C -2.81400000 -1.86900000 0.86900000

H -2.95600000 -2.10400000 1.94000000

H -3.76700000 -1.47300000 0.47000000

S -1.58600000 -0.48800000 0.76200000

H -3.13400000 -3.99300000 0.33900000

H -1.35500000 -7.56000000 0.86600000

C -0.41000000 -7.47000000 1.49300000

H 0.36800000 -7.95800000 0.87800000

C -0.10700000 -5.97200000 1.62400000

H -0.85200000 -5.46500000 2.26100000

H -0.11100000 -5.46900000 0.64100000

S 1.56300000 -5.70400000 2.38200000

H -0.53700000 -7.96300000 2.48600000

H -2.21800000 -1.10500000 8.44400000

C -1.19800000 -1.52200000 8.09500000

H -1.41900000 -2.53700000 7.72100000

C -0.61000000 -0.68600000 6.95300000

H -0.55700000 0.38600000 7.22500000

H -1.21500000 -0.79200000 6.03800000

S 1.11200000 -1.27200000 6.56000000

H -0.55200000 -1.56000000 8.99200000

H 3.63300000 0.15700000 -1.02400000

C 4.63000000 0.53100000 -0.66700000

H 4.86400000 1.39600000 -1.32300000

C 4.57800000 1.00900000 0.79100000

H 3.63100000 1.52600000 1.03500000

H 5.38700000 1.75300000 0.93200000

S 4.86600000 -0.27500000 2.09300000

H 5.37000000 -0.27100000 -0.86200000

Fe 0.23400000 -1.28000000 1.85200000

S 1.26600000 0.38400000 2.92000000

Fe 2.80600000 -1.20100000 2.24200000

S 1.83600000 -2.30400000 0.50400000

Fe 1.54900000 -3.45100000 2.54400000

S -0.47500000 -2.76700000 3.47700000

Fe 1.18800000 -1.52100000 4.29100000

S 3.15300000 -2.72800000 3.91000000

Hip1-Red-L

H -2.23700 -2.96300 -0.92600

C -2.40900 -3.16100 0.18400

H -1.42000 -3.47500 0.56700

C -2.83700 -1.86500 0.85600

H -2.96900 -2.08800 1.93000

H -3.79900 -1.49200 0.45500

S -1.63800 -0.46000 0.71700

H -3.13500 -3.99200 0.33800

H -1.35600 -7.56500 0.86500

C -0.41100 -7.48400 1.48900

H 0.35900 -7.98600 0.87500

C -0.07900 -5.99500 1.60900

H -0.80700 -5.46800 2.24900

H -0.07500 -5.49500 0.62400

S 1.60600 -5.77900 2.35700

H -0.53800 -7.97000 2.48400

H -2.22000 -1.10700 8.44800

C -1.20100 -1.52000 8.10100

H -1.41600 -2.53100 7.71100

C -0.60900 -0.66300 6.98000

H -0.56300 0.40400 7.27000

H -1.19900 -0.75600 6.05400

S 1.12000 -1.23800 6.61300

H -0.55500 -1.56000 8.99600

H 3.63600 0.15900 -1.03200

C 4.63600 0.53900 -0.69100

H 4.86900 1.38600 -1.36900

C 4.60100 1.05500 0.75500

H 3.63700 1.53200 1.01600

H 5.37600 1.83800 0.86100

S 4.98100 -0.18700 2.08000

H 5.37300 -0.26800 -0.87300

Fe 0.18600 -1.23900 1.79800

S 1.28700 0.38300 2.91300

Fe 2.93600 -1.14000 2.21400

S 1.87600 -2.28200 0.51300

Fe 1.59400 -3.53300 2.53200

S -0.43500 -2.76200 3.46200

Fe 1.19100 -1.49100 4.35900

S 3.16800 -2.70700 3.91000

Hip2-Ox-L

H 3.47700000 1.90200000 -2.13200000

C 2.61100000 1.70000000 -2.86200000

H 2.04900000 0.86000000 -2.42100000

C 1.73100000 2.94600000 -3.01600000

H 1.04300000 2.76200000 -3.85900000

H 2.32700000 3.84500000 -3.26700000

S 0.67300000 3.44600000 -1.57000000

H 2.99300000 1.42000000 -3.86300000

H 3.90900000 -2.38900000 -4.20000000

C 3.07500000 -3.01700000 -3.75700000

H 3.56900000 -3.67700000 -3.02200000

C 2.05200000 -2.08800000 -3.08100000

H 1.53100000 -1.46700000 -3.82600000

H 2.51100000 -1.40900000 -2.34300000

S 0.72100000 -3.06000000 -2.18500000

H 2.59300000 -3.58400000 -4.56800000

H -5.11700000 1.43400000 -6.60500000

C -4.96200000 0.55100000 -5.89500000

H -3.97400000 0.11800000 -6.13500000

C -4.95000000 1.08300000 -4.46700000

H -5.91100000 1.56300000 -4.22400000

H -4.14300000 1.82200000 -4.31700000

S -4.70800000 -0.30800000 -3.26800000

H -5.77100000 -0.15500000 -6.14900000

H -0.22700000 1.27900000 3.42700000

C -1.09200000 0.94900000 4.05600000

H -1.10400000 1.63500000 4.93000000

C -2.43400000 1.07000000 3.34500000

H -2.50500000 1.95300000 2.69500000

H -3.22500000 1.16800000 4.11300000

S -2.90900000 -0.38900000 2.33100000

H -0.87800000 -0.04900000 4.41800000

Fe -2.81000000 0.17200000 -2.21000000

Fe -1.87000000 -0.00500000 0.42300000

Fe -0.62000000 1.64900000 -1.42600000

Fe -0.53900000 -1.30800000 -1.61900000

S -2.58200000 -1.75700000 -0.89700000

S -2.67600000 1.93200000 -0.63900000

S 0.37800000 0.09500000 0.07800000

S -0.85000000 0.30400000 -3.32400000

Hip2-Ox-S

H 3.47700 1.89800 -2.13100

C 2.61300 1.68600 -2.86000

H 2.06900 0.83200 -2.42200

C 1.70000 2.90700 -3.00100

H 1.04500 2.72900 -3.87200

H 2.27400 3.83300 -3.20200

S 0.58700 3.30000 -1.57500

H 2.99400 1.41400 -3.86200

H 3.90700 -2.38800 -4.20100

C 3.06800 -3.00700 -3.75600

H 3.55300 -3.66100 -3.00800

C 2.05800 -2.04600 -3.10500

H 1.55600 -1.43500 -3.87000

H 2.53500 -1.35800 -2.38800

S 0.68800 -2.92800 -2.19600

H 2.58900 -3.57900 -4.56700

H -5.11300 1.43600 -6.59500

C -4.95400 0.54800 -5.88200

H -3.97500 0.10800 -6.14900

C -4.87900 1.08500 -4.45200

H -5.80800 1.61000 -4.17800

H -4.03500 1.78800 -4.33900

S -4.65300 -0.28400 -3.21600

H -5.76800 -0.15700 -6.13900

H -0.23500 1.27600 3.41700

C -1.11800 0.93800 4.01600

H -1.17600 1.63600 4.87700

C -2.43500 1.03500 3.25400

H -2.50000 1.92500 2.61300

H -3.25300 1.10700 3.99600

S -2.87600 -0.40800 2.19900

H -0.89300 -0.05400 4.40000

Fe -2.76900 0.12300 -2.08900

Fe -1.91400 -0.02600 0.27000

Fe -0.62700 1.45000 -1.35300

Fe -0.53300 -1.16300 -1.57500

S -2.58100 -1.79800 -0.88900

S -2.71300 1.87700 -0.59800

S 0.30200 0.06200 0.20900

S -0.92900 0.30300 -3.31700

Hip2-Red-L

H 3.48000000 1.90000000 -2.13200000

C 2.61300000 1.68800000 -2.86200000

H 2.06400000 0.84200000 -2.41300000

C 1.70900000 2.91800000 -2.99000000

H 1.00100000 2.70100000 -3.81000000

H 2.28900000 3.81900000 -3.28100000

S 0.70800000 3.38000000 -1.50800000

H 2.99400000 1.41500000 -3.86200000

H 3.90900000 -2.38800000 -4.20300000

C 3.07000000 -3.01200000 -3.75400000

H 3.56600000 -3.67100000 -3.01800000

C 2.04900000 -2.08300000 -3.07900000

H 1.53100000 -1.46200000 -3.82600000

H 2.52200000 -1.39500000 -2.35700000

S 0.72400000 -3.03500000 -2.17400000

H 2.59000000 -3.58100000 -4.56600000

H -5.11700000 1.43700000 -6.60200000

C -4.96200000 0.55100000 -5.88100000

H -3.97100000 0.12000000 -6.11700000

C -4.95100000 1.08200000 -4.45300000

H -5.89900000 1.60100000 -4.23000000

H -4.12100000 1.79500000 -4.30200000

S -4.74900000 -0.32200000 -3.26600000

H -5.77000000 -0.15500000 -6.14200000

H -0.21700000 1.28100000 3.44400000

C -1.07300000 0.96100000 4.08500000

H -1.04900000 1.63100000 4.97300000

C -2.42800000 1.12700000 3.39800000

H -2.43600000 1.96000000 2.68000000

H -3.19000000 1.35000000 4.17200000

S -3.01700000 -0.35800000 2.49700000

H -0.87000000 -0.04500000 4.42900000

Fe -0.58700000 1.52700000 -1.27700000

Fe -0.54100000 -1.24900000 -1.51900000

Fe -2.84400000 0.14900000 -2.11500000

Fe -1.98100000 -0.05300000 0.52400000

S -2.60700000 -1.82300000 -0.84300000

S -2.69000000 1.93200000 -0.55200000

S 0.30800000 0.03000000 0.31800000

S -0.89700000 0.30500000 -3.25900000

Hip2-Red-S

H 3.48000 1.89800 -2.13100

C 2.61200 1.68200 -2.85900

H 2.07500 0.82900 -2.41100

C 1.69100 2.90000 -2.97800

H 0.99700 2.68500 -3.81100

H 2.25900 3.81500 -3.24500

S 0.65700 3.31700 -1.50800

H 2.99300 1.41300 -3.86100

H 3.90700 -2.38700 -4.20200

C 3.06400 -3.00700 -3.75100

H 3.55700 -3.66400 -3.01100

C 2.04700 -2.06600 -3.08400

H 1.53900 -1.44500 -3.83700

H 2.52600 -1.37900 -2.36600

S 0.70100 -2.98400 -2.17800

H 2.58700 -3.57900 -4.56400

H -5.11500 1.43700 -6.59800

C -4.95700 0.55000 -5.87300

H -3.97000 0.11700 -6.12100

C -4.92200 1.08100 -4.44200

H -5.86700 1.59800 -4.20400

H -4.09300 1.79800 -4.31100

S -4.69000 -0.31000 -3.24100

H -5.76800 -0.15600 -6.13600

H -0.22600 1.27800 3.43200

C -1.09900 0.95100 4.04800

H -1.12000 1.63700 4.92300

C -2.43200 1.07700 3.31000

H -2.44900 1.93000 2.61500

H -3.22500 1.25300 4.06600

S -2.94300 -0.40000 2.35000

H -0.88300 -0.04900 4.41300

Fe -0.61500 1.44500 -1.27500

Fe -0.55700 -1.18900 -1.51600

Fe -2.77100 0.14100 -2.08200

Fe -1.91900 -0.04100 0.38100

S -2.58200 -1.84300 -0.85900

S -2.67800 1.93800 -0.56300

S 0.34600 0.02500 0.30200

S -0.88300 0.30800 -3.28600

# References

1. Ryde, U. QM/MM calculations on proteins. *Methods Enzymol.* **577**, 119–158 (2016).

2. Senn, H. M. & Thiel, W. QM/MM methods for biomolecular systems. *Angew. Chemie - Int. Ed.* **48**, 1198–1229 (2009).

3. Reuter, N., Dejaegere, A., Maigret, B. & Karplus, M. Frontier bonds in QM/MM methods: A comparison of different approaches. *J. Phys. Chem. A* **104**, 1720–1735 (2000).

4. Ryde, U. The coordination of the catalytic zinc in alcohol dehydrogenase studied by combined quantum-chemical and molecular mechanics calculations. *J. Comput. Aided. Mol. Des.* **10**, 153–164 (1996).

5. Hu, L., Söderhjelm, P. & Ryde, U. On the convergence of QM/MM energies. *J. Chem. Theory Comput.* **7**, 761–777 (2011).

6. Case, D. A. *et al.* Amber 14, University of California: at (2014).

7. Ryde, U. & Olsson, M. H. M. Structure, strain, and reorganization energy of blue copper models in the protein. *Int. J. Quantum Chem.* **81**, 335–347 (2001).

8. Cao, L. & Ryde, U. On the difference between additive and subtractive QM/MM calculations. *Front. Chem.* **6**, 89 (2018).
